# Perturbed Lipidomic Profiles in Rats With Chronic Cerebral Ischemia Are Regulated by Xiao-Xu-Ming Decoction

**DOI:** 10.3389/fphar.2019.00264

**Published:** 2019-03-19

**Authors:** Zhixin Jia, Cai Tie, Caihong Wang, Caisheng Wu, Jinlan Zhang

**Affiliations:** State Key Laboratory of Bioactive Substance and Function of Natural Medicines, Institute of Materia Medica, Chinese Academy of Medical Sciences and Peking Union Medical College, Beijing, China

**Keywords:** chronic cerebral ischemia, lipidomics, lipid metabolic network, potential biomarkers, Xiao-Xu-Ming decoction

## Abstract

Chronic cerebral ischemia (CCI) is a serious human health condition with lacking therapeutic agents. Moreover, its mechanism of action remains elusive, and thus novel treatment options are required. Lipid metabolism disorder are closely related to CCI. In this study, a CCI-rats model was established by the permanent occlusion of rat bilateral common carotid arteries, and then the rats were treated with a Xiao-Xu-Ming decoction (XXMD). Lipidomic profiling was conducted in both plasma and brain o determine the effects of the injury and therapy on lipid metabolism. Sphingolipid (particularly long acyl chain and total ceramides), glyceryl phosphatide, and glyceride profiles significantly changed in the brain after model induction and again after dosing. A total of 35 potential biomarkers were found in the brain and four were found in the plasma, representing both CCI injury and XXMD action. Correlations between endogenous lipids and exogenous XXMD compounds were analyzed using linear regression. Two exogenous compounds (cimifugin and 5-*O*-methylvisamminol) in the brain and 17 exogenous compounds in the plasma, which may represent the active constituents in XXMD, were significantly associated with lipid metabolism. This study provides a new perspective on the potential mechanism of CCI and its treatment with XXMD, as well as on discovering effective components in traditional Chinese medicines.

## Introduction

Cerebral ischemia is one of the top three human physical and mental health risks and is associated with high mortality and morbidity rates. The incidence of cerebral ischemia has been rising annually, affecting younger patients as well because of dietary changes and an accelerated pace of life (Meulman et al., 2015). Chronic cerebral ischemia (CCI) is a type of nervous system damage caused by the prolonged restriction of the blood supply to the brain tissue below a certain threshold. CCI is the pathological basis for acute and chronic ischemic cerebrovascular diseases (Farkas and Luiten, 2001). Insufficient blood supply associated with CCI is the first step in the progression of vascular dementia (VD) and Alzheimer’s disease (AD) (de la Torre, 2004).

Lipid metabolism disorder injures the central nervous system (CNS) and are associated with cerebral ischemia, VD, and AD (Ramesh et al., 2012). CNS is lipid-rich, and lipids are known to form cellular structures such as phospholipids and store energy, e.g., in the form of triglycerides (TGs). Certain lipids are precursors of various secondary messengers, including arachidonic acid (ArAc), docosahexaenoic acid, ceramides (Cers), and diacylglycerol (DG) (Wenk, 2005). Cerebral ischemia leads to an insufficient energy supply and causes the release of neurotransmitters such as dopamine and glutamic acid (Adibhatla and Hatcher, 2004). When the glutamic acid receptor is overexcited, it increases intracellular Ca^2+^ and activates phospholipases A2, C and D, and sphingomyelinase. These enzymes hydrolyze membrane phospholipids and release secondary messengers (Adibhatla and Hatcher, 2004) including DG, phosphatidic acid (PA), lyso-PA, and ArAc. Accumulation of inflammatory cytokines, such as tumor necrosis factor-alpha and interleukin-1, also increases phospholipase activity (Wang and Shuaib, 2002). Cers are generated by sphingomyelinase after ArAc stimulation and induce apoptosis and tissue damage by inhibiting mitochondrial electron transport and via release of cytochrome c (Wenk, 2005). In stroke patients, total serum cholesterol and TG levels are associated with cerebral ischemia. Medicinal therapy is significantly correlated with the regulation of lipid metabolism regulation (Fuentes et al., 2009). Moreover, recent studies have shown that the sphingolipid and phospholipid profiles in the white matter and ectocinerea are markedly altered in VD patients. Therefore, lipidomic studies are important (Lam et al., 2014).

The mechanism underlying the cerebral ischemia-caused injury remains unclear. Establishing preclinical CCI animal models helps determine the pathogenesis and pathological characteristics of CCI. The two-vessel occlusion (2VO) rat model was developed by de la Terre in 1992 and is now widely used as a CCI animal model for the evaluation of the efficacy of therapeutic agents. Briefly, the bilateral common carotid arteries of the rats are exposed and carefully separated from their carotid sheaths, avoiding injury to the vagus nerve, and then ligated with surgical silk. Behavioral experiments are carried out in rats for validation.

Xiao-Xu-Ming decoction (XXMD) is a traditional Chinese medicine (TCM), first recorded in *Bei Ji Qian Jin Yao Fang* written by Simiao Sun at the time of the Tang Dynasty (Wang Y. et al., 2012). XXMD has been selected to be included in the first batch of classical prescriptions (#32) by the Chinese government. This formulation consists of 12 crude herbs, and its clinical efficacy has been reported for stroke patients (Fu et al., 2013). Besides, modern pharmacological studies have demonstrated that XXMD shows neuroprotective effects (Zhu et al., 2010). The active fractions in Chinese medicines contains pharmacologically active ingredients used in clinical applications. Previous studies have shown that the active fraction of XXMD is eluted from a macroporous resin with 40% ethanol elution (Fu et al., 2013). The composition of the active XXMD fraction and its metabolic characteristics have been investigated in our previous study (Wang Y. et al., 2012).

A CCI rat model was established using the 2VO method in the present study to elucidate the effects of cerebral ischemia and its treatment on lipid profiles. A sham operation, an untreated model, and a model treated with the active XXMD fraction were used. A Morris water maze test was performed, and biochemical parameters, and staining characteristics were evaluated to test model effectiveness. A targeted comprehensive lipidomic platform was used to characterize the lipid profiles of the brain and plasma in all three groups. The lipid profiles significantly changed after the 2VO model was induced and again after oral administration of the active XXMD fraction. Multivariate statistical analysis was performed to identify potential biomarkers associated with the injury in the CCI model as well as with its improvement following administration of the active XXMD fraction. The components and metabolites of the active XXMD fraction in rat brain and plasma were identified by high-performance liquid chromatography tandem high-resolution mass spectrometry (HPLC-HRMS/MS) and statistically correlated with potential lipid biomarkers by linear regression analysis. In the present study, the mechanisms of CCI injury, its treatment with XXMD, and the identity of its active compounds were investigated from a lipidomics perspective.

## Materials and Methods

### Materials

#### Animals

Male Wistar rats weighing 350 ± 25 g were purchased from Vital River Laboratories (Beijing, China) and maintained under a 12-h light/dark cycle prior to the experiment. All rats were housed for 2 weeks to allow acclimatization before treatment.

#### Standards and Reagents

All lipid standards were purchased from the Avanti Company (Alabaster, AL, United States). Please refer to Supplementary Table S1.

Methanol (ultra resi-analyzed grade) and methyl tertiary butyl ether (MTBE, HPLC grade) were purchased from Avantor Performance Materials, LLC (Center Valley, PA, United States). Formic acid (analytical grade) was purchased from the Tedia Company, Inc. (Fairfield, OH, United States). Ammonium formate (purity > 99.99%) was purchased from Sigma-Aldrich Corp., St. Louis, MO, United States. Ultra-pure water was prepared using a Milli-Q purification system (EMD Millipore, Burlington, MA, United States).

Superoxide dismutase (SOD) and malondialdehyde (MDA) assay kits were obtained from the Nanjing Jiancheng Bioengineering Institute (Nanjing, China). A trace bicinchoninic acid (BCA) protein quantification kit was purchased from the Wuhan Boshide Biological Engineering Company, Wuhan, China.

The XXMD formulation consists of 12 crude herbs, including divaricate saposhnikovia root, baical skullcap root, debark peony root, licorice root, fresh ginger, mealy fangji root, ginseng, cassia twig, bitter apricot seed, ephedra, sichuan lovage rhizome, and prepared common monkshood branched root, mixed at a ratio of 3:3:3:3:3:3:6:6:6:9:9:9 on a dry weight basis, respectively. All materials were purchased from Tong-rentang, Ltd. (Beijing, China), a famous and time-honored brand in the TCM industry, and were identified by Dr. Lin Ma (Associate Professor, Institute of Materia Medica, Chinese Academy of Medical Sciences & Peking Union Medical College) to be the root of *Saposhnikovia divaricata* (Turcz.) Schischk., the root of *Scutellaria baicalensis* Georgi, the root of *Paeonia lactiflora* Pall., the root of *Glycyrrhiza uralensis* Fisch. ex DC., the root of *Zingiber officinale* Roscoe, the root of *Stephania tetrandra* S. Moore, the root of *Panax ginseng* C.A.Mey., the twig of *Cinnamomum cassia* (L.) J.Presl, the seed of *Prunus armeniaca* L. var. *ansu* Maxim., the stem of *Ephedra sinica* Staph, the root of *Ligusticum chuanxiong* Hort., and the root of *Aconitum carmichaeli* Debeaux. All the voucher specimens (numbered IMM-SD-200911, IMM-SB-200916, IMM-PL-200932, IMM-GU-200935, IMM-ZO-200919, IMM-ST-200925, IMM-PG-200905, IMM-CC-200906, IMM-PA-200913, IMM-ES-200939, IMM-LC-200941, and IMM-AC-200951, respectively) have been deposited at the Department of Pharmaceutical Analysis, Institute of Materia Medica, Chinese Academy of Medical Sciences & Peking Union Medical College (Beijing, China). The detailed information on the herbs (Chinese/English/Latin names, families, and used parts) is listed in Supplementary Table S1 (Rui et al., 2014).

The active XXMD fraction was screened by Wang et al. (2011), and shown to exert a similar pharmacological effect to that of XXMD in treating theoplegia and its sequelae. In our previous studies, the chemical fingerprints of the active XXMD fraction were studied, and its active constituents were identified. In addition, their relative quantities were determined (Wang et al., 2014, 2016). The mass fingerprints and chemical structures of the active constituents are shown in Supplementary Figure S1. The contents are shown in Supplementary Table S2.

#### Instruments

The Morris water maze (Type DMS-2) was developed by the Institute of Materia Medica, Chinese Academy of Medical Sciences & Peking Union Medical College, Beijing, China. Sphingolipids were quantified with an Agilent 6410B triple quadrupole mass spectrometer (Agilent Technologies, Inc., Santa Clara, CA, United States) consisting of a triple quadrupole MS analyzer and an electrospray ionization (ESI) interface in positive ionization mode and an Agilent 1200 RRLC system. A Thermo Fisher Scientific LTQ FT connected to a Thermo Fisher Scientific Surveyor LC Plus system with an electrospray ionization (ESI) interface was used to quantify other lipids (Thermo Fisher Scientific, Waltham, MA, United States). A plate reader (Tecan Sunrise; Gemini BV, Apeldoorn, Netherlands) was used for the biochemical tests.

### Methods

#### Morris Water Maze

The Morris water maze was first developed by Morris (1984). In our study, a type DMS-2 water maze was developed by the Institute of Materia Medica, Chinese Academy of Medical Sciences & Peking Union Medical College. The cylindrical water tank in the Morris water maze was 120 cm in diameter and 50 cm in height. It was filled with water at 22–25°C to a depth of 30 cm, which and covered a black platform of 10 cm in diameter. The tank was divided into four equal quadrants labeled 1–4. The black platform was 1 cm below the water surface.

##### Navigation training trial

Within each 12-h period, the rats were allowed one attempt to find the hidden platform. In the morning, each rat was gently placed in the water, in the middle of the first quadrant, facing the maze side wall. The time required for each rat to reach the platform was recorded. The maximum trial duration was 60 s. The rats were then guided to the platform by hand and kept there for 20 s for reinforcement. Those capable of finding the platform on their own were also kept there for 20 s before the end of the training session. In the afternoon, the rats were placed into the maze in the middle of the fourth quadrant. All the other test conditions were the same as described above.

##### Space exploration trial

The platform was removed, and the rats were gently placed into the water in the middle of the first quadrant. The arrival time was recorded when the rat first reached the position where the platform was located. If the rat did not reach this position, then its dwell time in the targeted quadrant was recorded instead. These data were used to analyze the memory behavior (Morris, 1984).

The laboratory environment, including the light intensity and object placement, remained constant throughout the trial period. A maximum of two experimenters were allowed in the laboratory at a time.

#### Animals Experiments

Animal experimentation began after 2 weeks of acclimation. CCI was induced in rats using 2VO according to the method of Wakita et al. (1995). Wistar rats were fasted for 12 h and deprived of water for 4 h before surgery. They were then anesthetized with chloral hydrate [400 mg⋅kg^−1^, intraperitoneally (i.p.)], placed in a supine position on a heating pad to maintain their body temperature at 37.5 ± 0.5°C, and disinfected with an alcohol prep pad. The skin in the middle of the right side of the neck was cut, and the muscle tissue was bluntly separated to avoid injury to the vagus nerve and weasand. The bilateral common carotid arteries of the rats were exposed and carefully separated from the carotid sheaths. The arteries were then ligated with 5-0-type surgical silk in ischemic rats but not in sham operation rats. The incisions were sutured with 0-type surgical silk.

The navigation training trial took place on days 22–26 after surgery (Wang Y.H. et al., 2012) to verify the successful development of the 2VO model. The screening criteria were as follows: (average time - reference time)/average time >0.2, where the reference time was the average arrival time of the rats from the sham operation group, and the average time was the average arrival time of the rats from the model group. Animals meeting these criteria were considered to be successfully modeled.

The successfully modeled rats were randomly divided into a model group (*n* = 10) and a dosed model group (*n* = 9). The sham operation group (*n* = 10) served as a control, and all three groups were subjected to lipidomic profiling. The rats in the dosed model group were orally administered the active XXMD fraction for 1 month (0.15 g⋅kg^−1^, days 27–56 after surgery) (Wang Y.H. et al., 2012). Three time points (0.5, 2.5, and 8 h after the last oral dose) were set, and three animals were assessed at each time point. The other animals in the dose group and those in the other groups were sacrificed 12 h after the last oral XXMD administration. The Morris water maze test was carried out during the last 5 days of dosing (days 52–56 after surgery). The navigation training trial lasted for 5 days. The space exploration trial was run after the navigation training trial, on day 56. On day 57, the animals were sacrificed after the second Morris water maze test. Brain tissue and plasma samples were collected as described below.

Animal care and experimental procedures were reviewed and approved by the Institutional Animal Care and Use Committee of the Chinese Academy of Medical Sciences & Peking Union Medical College. All procedures were carried out in accordance with the approved guidelines.

#### Sample Collection

##### Brain tissue and plasma

Animals were euthanized by stunning and decapitation. They were then rapidly dissected. Blood was collected in a centrifuge tube containing heparin. Plasma was collected by centrifugation (4°C, 4,500 × *g*, 10 min). The whole brain was taken out, rinsed with normal saline, and quickly frozen in liquid nitrogen. A total of 6 mL normal saline was added to each piece of brain tissue for later homogenization. All samples were stored at −80°C until biochemical and lipid analysis.

##### Fixing of brain tissue

The rats were anesthetized by using chloral hydrate (400 mg kg^−1^, i.p.). The abdomen was then cut to expose the heart. A blunt needle was inserted into the ascending aorta from the right atrium. The auricula dextra was cut off and irrigated with normal saline until the liver turned white. Around 10% formaldehyde was then applied until the tail stiffened. The integrated brain tissue was carefully excised and fixed in 10% formaldehyde. The fixed brain was sectioned for Klüver–Barrera and Nissl staining.

#### Pathological Detection

Nissl staining was employed to observe the structures of the hippocampus and cortical neurons. Klüver–Barrera staining was performed to observe the white matter. Section preparation and staining were performed by the Beijing Xuebang Technology Co., Ltd., Beijing, China.

#### Protein Quantification

Protein was quantified by the BCA method (Smith et al., 1985; Masukawa et al., 2009) using a trace BCA protein quantification kit. A Tecan Sunrise (Gemini B.V.) plate reader was used to detect the absorbance according to the manufacturer’s instructions.

#### Biochemical Analysis

Brain tissue and plasma were thawed at 4°C. SOD and MDA levels were measured according to the manufacturer’s instructions for the kits. SOD was quantified by the xanthine oxidase method and MDA was measured by the thiobarbituric acid method. A Tecan Sunrise plate reader (Gemini BV) was used to measure the absorbance according to the manufacturer’s instructions.

#### Lipidomic Profiling

Lipids were analyzed using a targeted lipidomic platform. A flowchart detailing this process is shown in Supplementary Figure S2. Sphingolipids that were relatively low in abundance were analyzed via HPLC coupled to triple quadrupole MS. A total of 86 compounds were detected within 25 min. Other, relatively more abundant, lipids were analyzed by HPLC coupled to Fourier-transform ion cyclotron resonance (FTICR) MS. A total of 589 compounds were detected within 30 min, including seven lysophosphatidic acids, 24 lysophosphatidylcholines (LPCs), 10 lysophosphatidylethanolamines (LPEs), 6 lysophosphatidylglycerols, seven lipopolysaccharides, 63 PAs, 50 phosphatidylcholines (PCs), 62 phosphatidylethanolamines (PEs), 43 phosphatidylglycerols, 26 polysaccharides (PSs), 28 sphingomyelins (SMs), 2 monoglycerides, 66 DGs, and 195 TGs. A total of 221 compounds were detected in brain tissue and 218 were detected in plasma using the Lipid Data Analyzer (LDA) software (Graz University of Technology, Graz, Austria) associated with this platform. Detailed information is provided in Supplementary Table S2.

Sphingolipids were identified by comparing their retention times with those of authentic standards and were quantified using standard curves plotted with internal standards (shown in Supplementary Table S6). Other lipids were quantified using LDA (Hartler et al., 2011). The protein content of the brain tissue was used to normalize the concentration of the lipids detected.

#### Metabolite Identification and Detection

Xiao-Xu-Ming decoction metabolites were identified and characterized in the brain tissue and plasma using the mass spectral trees similarity filter (MTSF) technique (Jia et al., 2014; Wang et al., 2014). Mass spectral trees were constructed for the components detected in the active fraction to build a library using full scan mass spectra as the stem and multiple-stage mass spectra as the branches. Metabolites were discovered and identified by comparing their mass spectral trees with those of the known compounds in the library. Active exogenous components were determined semi-quantitatively by HPLC-HRMS. Sample pretreatment and analytical methods are described in Supplementary Table S3.

#### Statistical Analysis

Differences in lipid profiles between groups were identified using SPSS v. 18.0 (IBM Corp., Armonk, NY, United States). *P* < 0.05 indicated statistical significance. Variables were separated into two groups using a normality test. Those fitting a normal distribution were evaluated by one-way analysis of variance. No normally distributed data were evaluated by a Mann–Whitney *U*-test. The data were exported to SIMCA-P+ 12.0.1 (Umetrics AB, Umeå, Sweden) for multivariate statistical analyses. Principal component analysis (PCA) was used for visual group discrimination. Orthogonal partial least squares discriminant analysis (OPLS-DA) was used to identify potential biomarkers and perform T-predictions. Data in each group were generated using a normalized, mean-centered unit variance scale. Potential biomarkers were selected according to the following three criteria: the variable importance in the projection must be >1; the jack-knife uncertainty bar must exclude zero; and the absolute value of Pcorr in the S-plot must be >0.58. T-prediction was used to compare the recovery tendency between the dosed model and model groups. Correlation analysis between the potential lipid biomarker concentrations and the peak areas of the base XXMD material was performed using SPSS for each of the three time points set. A Pearson correlation analysis was then run for these time points. The absolute values of the relative coefficients for these peak areas were set to >0.997.

## Results

### Verification of the CCI Model and the Effect of XXMD

A water maze test, biochemical parameters, and histopathological analyses were used to evaluate the stability of the 2VO model and the curative potential of XXMD (Ji et al., 2010).

**FIGURE 1:**
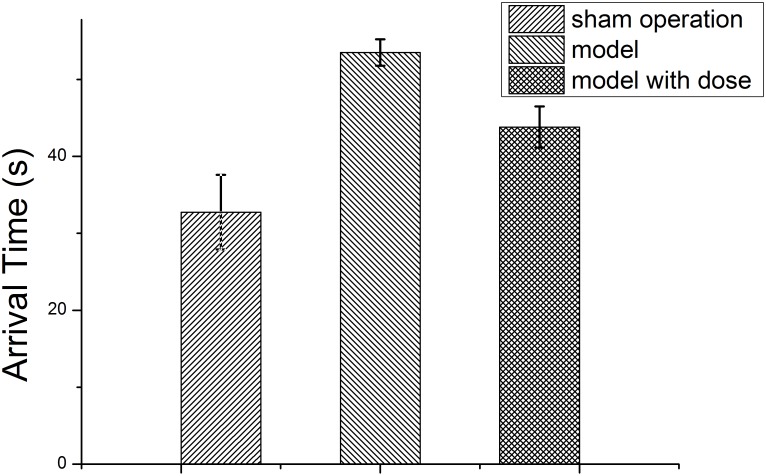
Bar chart of the water maze data.

The rat arrival times in the water maze test are listed in Table 1. The model and sham operation groups had the longest and shortest arrival times, respectively, and both were significantly different from that of the dosed model group (*P* < 0.01). However, there was no significant difference between the arrival time of the sham operation group and that of the dosed model group. A bar chart with the water maze data is shown in Figure 1.

**Table 1:** Data from the water maze and the biochemical analyses.

Groups	Arrival time (s)	MDA in brain (nM/mg Pr)	MDA in plasma (nml/mL)	SOD in brain (U/mg Pr)	SOD in plasma (U/mL)
Sham operation	32.74 ± 4.9	1.87 ± 0.15	3.65 ± 0.42	3.30 ± 0.36	37.81 ± 0.90
Model	53.49 ± 1.7	2.48 ± 0.39	5.45 ± 0.65	2.82 ± 0.22	34.80 ± 2.6
Model with dose	43.81 ± 2.7	1.94 ± 0.13	1.09 ± 0.42	3.13 ± 0.13	52.5 ± 10

**Comparison**	***P*-value**				

Sham operation vs. Model	0.002	0.012	0.001	0.034	0.039
Model vs. Model with dose	0.001	0.020	0.000	0.029	0.005
Sham operation vs. Model with dose	0.050	0.303	0.622	0.168	0.059

*Arrival time, MDA, and SOD values are expressed asaverage ± SD.*

The results of the biochemical data analysis are shown in Table 1. Compared with that in the sham operation group, MDA was significantly higher in the model group (*P* < 0.05) and significantly decreased (*P* < 0.05) after XXMD treatment in both brain tissue and plasma. SOD presented an opposite trend; however, no significant differences were observed between the sham operation and dosed model groups in terms of the brain and plasma SOD levels.

Histopathology of the brain tissue is shown in Supplementary Figure S3. Nissl staining showed that the hippocampus CA1 area and the cortex exhibited typical neuropathological changes in the 2VO model group. In the sham operation group, neurons were moderate in size, clear, and had a normal structure. However, neurons in the model group exhibited significant shrinkage, loss, and dark staining. These characteristics substantially improved in the dosed group. Klüver–Barrera staining showed that vacuolation and demyelinated fibers were more prominent in the white matter in the model group than in the sham operation group. The white matter health status markedly improved in the dosed model group.

These results indicate that the 2VO model was successfully established and stable and that XXMD significantly improved injury in terms of behavior, biochemical parameters, and histopathology.

### Targeted Organ and Plasma Lipidomic Profiling of Model CCI Rats and Those Dosed With XXMD

Brain tissue and plasma samples were analyzed using a targeted lipidomics platform to characterize their lipidomic profiles. The brain and plasma lipid concentrations were statistically processed to identify intergroup variations. A total of 89 lipids (nine classes) significantly changed in brain tissue, and 53 lipids (eight classes) significantly changed in the plasma in the model group compared with their levels in the sham operation group. A total of 116 lipids (11 classes) significantly changed in brain tissue, and 113 lipids (12 classes) significantly changed in the plasma after XXMD dosing. Bar charts of these compounds are shown in Supplementary Figures S4, S5.

**FIGURE 2:**
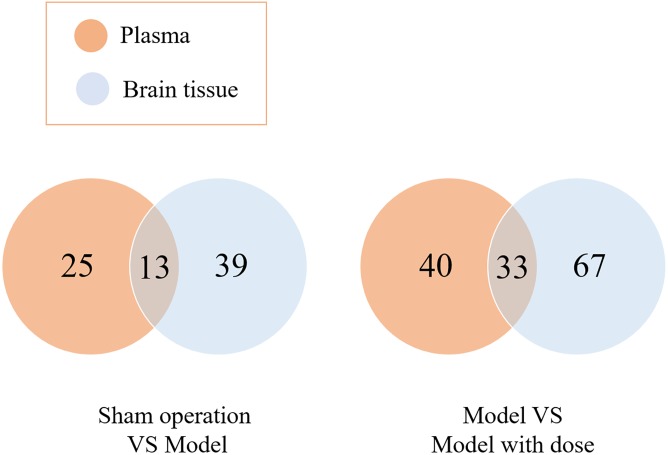
A Venn plot of blood biomarkers and the brain biomarkers.

Potential biomarkers were found by multivariate statistical analysis. A total of 52 potential biomarkers (eight classes) in brain tissue and 38 potential biomarkers (seven classes) in the plasma were associated with the model injury. A total of 100 potential biomarkers (10 classes) in the brain tissue and 73 potential biomarkers (10 classes) in the plasma indicated a restorative effect of XXMD. The trends are listed in Supplementary Table S5. A Venn diagram was used to demonstrate the relationships between the blood and brain biomarkers (Figure 2).

## Discussion

### Potential Biomarkers With Return Tendency

**FIGURE 3:**
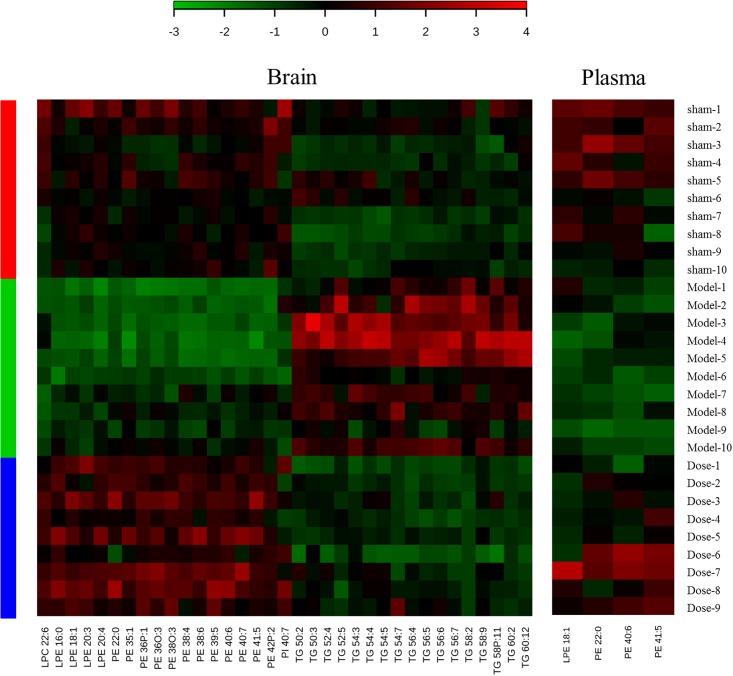
Heat maps of potential biomarkers with return tendency. The abscissa is the compound name, and the ordinate is the number of animals. Groups are listed on the left: red is the sham operation group, green is the model group, and blue is the dosed model group. Green patches represent a decrease, and red patches represent an increase. The color reference is provided by the scale bar at the top.

Further analysis identified potential biomarkers with return tendency. These biomarkers represented both the model injury and the therapeutic effect of XXMD. “Return tendency” describes the biomarkers that increased after the 2VO model induction and decreased after XXMD dosing or vice versa. A total of 35 return-tendency biomarkers were found in the brain tissue [LPCs, LPEs, PEs, phosphatidylinositols (PIs), and TGs], and four were found in the plasma (PEs and LPE). Heat maps of these compounds are shown in Figure 3. There were four potential return-tendency biomarkers (LPE 18:1, PE 22:0, PE 40:6, and PE 41:5) that exhibited the same trends in both brain tissue and plasma.

**FIGURE 4:**
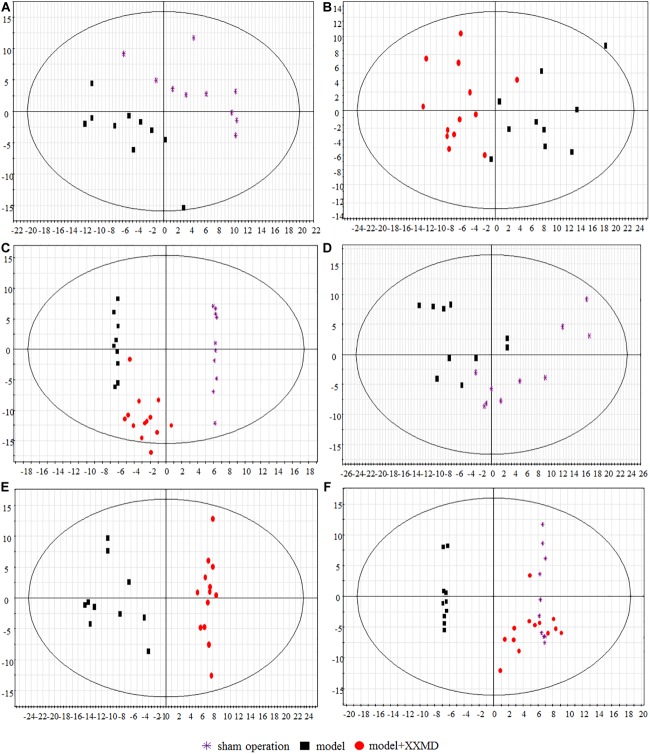
Score scatter plots of lipids by PCA and T-prediction by OPLS-DA. **(A)** PCA, model group and sham operation group with respect to plasma. **(B)** PCA, dosed model group and model group with respect to plasma. **(C)** OPLS-DA, T predicted score scatter plots of the dosed model group with respect to plasma. **(D)** PCA, model group and sham operation group with respect to brain tissue. **(E)** PCA, dosed model group and model group with respect to brain tissue. **(F)** OPLS-DA, T predicted score scatter plots of the dosed model group with respect to brain tissue.

Score scatter plots were generated by unsupervised PCA for visual group discrimination (Figure 4). The model and sham operation groups and the dosed model and model groups were distinguishable based on their measured brain and plasma lipid levels (Figure 4A,B,D,E). A T-prediction of OPLS-DA was used to illustrate the recovery trend of the dosed model group relative to the model group. To this end, SIMCA-P+ 12.0.1 was used. For brain tissue, the dosed model group located on the right side of the vertical axis was near the sham operation group but far from the model group (Figure 4F). Therefore, the lipids in the dosed model group recovered to the sham operation level and the active XXMD fraction significantly improved target organ injury. For the plasma, the model and sham operation groups exhibited tendencies resembling similar to those observed for the brain tissue (Figure 4C). The dosed model group diverged from the model group and approached the sham operation group. However, the recovery trend was not as clear as that in the brain tissue. These results clearly demonstrated that the brains and plasma in the 2VO rat model showed perturbed lipidomic profiles. The XXMD treatment restored the lipid profiles to normal levels. Moreover, the recovery effects were greater in the brain tissue (Figure 4F) than in the plasma (Figure 4C).

### Changes of Lipid Pathways

**FIGURE 5:**
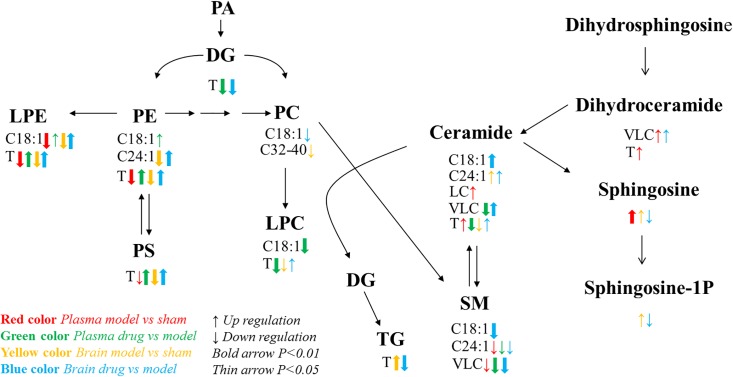
Lipid metabolic network and the variation tendency. Red arrows represent the comparison between the sham operation and model groups with respect to plasma; green arrows represent the comparison between the dosed model and model groups; yellow arrows represent the comparison between the sham operation and model groups with respect to brain tissue; blue arrows represent the comparison between the dosed model and model groups. Up arrows indicate an increase, and down arrows indicate a decrease. C18:1 indicates lipids with an 18:1 acyl chain; C24:1 indicates lipids with a 24:1 acyl chain; LC refers to long acyl chain lipids (≤20°C); and VLC refers to very long acyl chain lipids (≥20°C). T means total content.

Sphingolipids consist of long-chain (LC; ≤20°C) and very long chain (VLC; ≥22°C) classes (Pinto et al., 2014). Differences in the sphingolipid levels were significant among the groups (*P* < 0.05; Figure 5). Compared with those in the model group, the VLC Cer and VLC dihydroceramide (dhCer) levels were significantly higher, and the VLC SM level was significantly lower in the brain tissue of the dosed model group. In the plasma of the model group, the LC Cer and VLC and total dhCer levels were significantly higher than those in the sham operation group. These finding corroborates a report that stated that an increase in LC sphingolipids might reflect cellular damage (Wenk, 2005). VLC SMs were significantly lower in the model than in the sham group. In the plasma of the dosed model group, VLC and total Cers VLC SMs were lower than those in the model group. The trends in the in total Cers in the model and dosed model groups were the opposite between the brain tissue and plasma. One possible explanation is the release of Cers from the damaged brain tissue into the plasma.

Phosphatidylcholine (32–40 carbon atoms) significantly decreased in the brain tissue of the model group compared with the sham operation group. This observation is consistent with previous literature data (Kaddurah-Daouk et al., 2010). PC could effectively improve the learning memory in dysmnesic mice (Luo et al., 2011), and oral PC administration promoted synaptic plasticity in the hippocampus of young rats (Zhang et al., 2005). Thus, PCs may play a crucial role in brain tissue damage. Other cytomembrane phospholipids (LPEs, PEs, and PSs) also decreased. Therefore, phospholipids may be indicative of cell damage and brain tissue lesions (Luo et al., 2011). PE and PC levels were significantly lower in the plasma of patients with ischemic stroke than in those from the control group (Ma et al., 1998). In our study, XXMD dosing upregulated these lipids in the rat brains, suggesting that XXMD has a curative effect.

Triglycerides in the brain tissue were much higher in the model group than in the sham operation group. In the dosed model group, the active XXMD fraction significantly decreased TGs.

By combining previously reported findings on lipid metabolic pathways (Guan et al., 2011) with a detailed analysis of the present experimental data, we found that the C18:1 and C24:1 acyl chain lipid pathways, which involve sphingolipid, glyceryl phosphatide, and glyceride, were abnormal after brain injury and were clearly restored by XXMD. Especially noteworthy is the C18:1 acyl chain sphingolipid pathway in the brain tissue. Sphingosine and its downstream product sphingosine-1P both increased in the model group. XXMD upregulated the core compound Cer C18:1 and decreased its downstream products, namely, SM C18:1, sphingosine, and sphingosine-1P. These abnormal changes in brain tissue may contribute to the changes in oxidative stress parameters related to apoptosis (Wenk, 2005). The C24:1 acyl chain lipid pathway in brain tissue exhibited a trend similar to that of the C18:1 acyl chain lipid pathway. However, PE C24:1 decreased in the model group and increased after XXMD dosing. These results support the idea that both the 2VO model and the XXMD treatment had far greater effects on the target organ (brain) than they did on the plasma.

### Correlation Analysis Between Potential Lipid Biomarkers and Base XXMD Material

**FIGURE 6:**
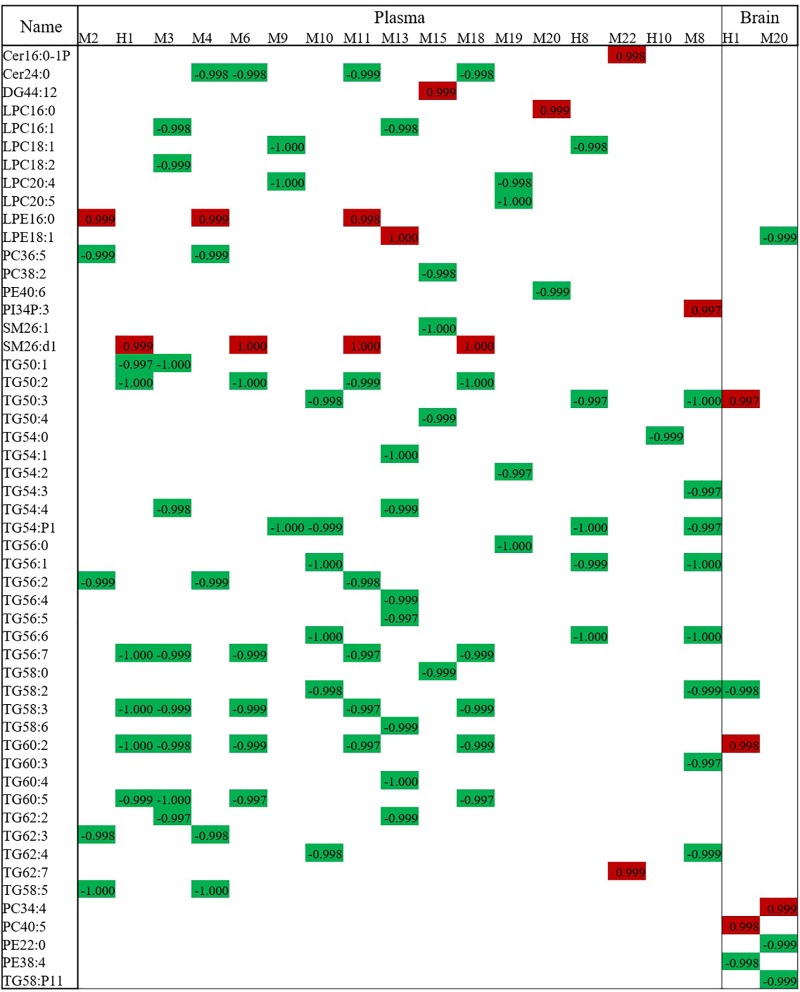
Correlation analysis of lipid biomarkers with exogenous metabolites. The numbers stand for the correlation coefficient. Red means positive correlation and green means negative correlation.

Components of XXMD and its metabolites, found in rats after oral administration, which correlated with XXMD effects, were analyzed in the dosed model. A total of 28 compounds were identified in the plasma (six parent compounds from the active XXMD fraction and 22 metabolites) and two compounds were identified in the brain (one parent compound from the active XXMD fraction and one metabolite) (see Supplementary Figure S6 and Supplementary Table S4 for structural information) (Wang et al., 2014). Attempts were made to correlate the peak areas of the active exogenous XXMD compounds with the concentrations of endogenous lipid biomarkers regulated by XXMD. Linear regression analysis was used to determine the associations between these compounds and the lipid biomarkers at three different time points. The highly correlated compounds and lipid biomarkers were termed compound pairs. The absolute values of the relative correlation coefficients for these pairs were >0.997. There were nine compound pairs in the brain tissue (corresponding to two exogenous compounds) and 95 compound pairs in the plasma (corresponding to 17 exogenous compounds). Compound pairs showing a linear correlation in the plasma included 47 lipids (two sphingolipids, one DG, two PCs, one PE, one PI, six LPCs, two LPEs, two SMs, and 30 TGs). M20 and M22 were positively correlated with most of the lipid biomarkers except LPE 16:0, LPE 18:1, PC 38:2, PE 40:6, PI 34:P3, SM 26:1, SM 26:d1, TG 50:4, and TG 58:0. LPE, PE, PI, and SM 26:d1 were positively correlated with most of the exogenous compounds except M20 and M22. Meanwhile most of the correlations of the pairs not mentioned above were negative. TGs were present in most of these lipids. Therefore, the curative effect of XXMD, then, may be influenced or induced by decreases in TGs. However, only two exogenous compounds, cimifugin (H1, PubChem CID: 441960) and 5-*O*-methylvisamminol (M20, PubChem CID: 441970), were detected in the brain tissue (Wang et al., 2014). They were associated with nine lipid biomarkers (one LPE, two PEs, two PCs, and four TGs). Figure 6 shows a bar chart of the correlation of these compounds with the biomarkers.

Significantly, more lipid compound pairs were found in the plasma than in the brain tissue. One possible explanation is that more exogenous compounds were detected in the plasma than in the brain tissue. However, metabolites such as cimifugin (H1) and 5-*O*-methylvisamminol (M20), which can cross the blood–brain barrier (BBB), may play important roles in regulating lipid metabolism and deserve further investigation.

## Conclusion

A previous study has demonstrated that lipid metabolism disorders are closely associated with cerebral ischemia but has mainly focused on the class of lipids or several single species (Lam et al., 2014). In this study, we performed a more detailed and deeper analysis in CCI rats via an established lipidomic platform. Both brain and plasma lipidomic profiles were characterized in CCI modeled rats and those dosed with the XXMD, revealing potential biomarkers and allowing building a lipid metabolism network (Figure 5). The regulation of lipid subclasses (such as TGs, PCs, PEs, PSs, and Cers) showed the same trends as those described in the literatures (Wenk, 2005; Luo et al., 2011). In addition, it is worth noting that the C18:1 and C24:1 acyl chain lipid metabolic pathways were also examined and the data were reflective of the entire network (especially in phospholipids and sphingolipids, shown in Figure 5), which is reported for the first time. Moreover, we analyzed each species of lipids and discovered four potentially crucial biomarkers with a return tendency in the plasma (LPE 18:1, PE 22:0, PE 40:6, and PE 41:5), which deserve further investigation from the point of view of mechanism of CCI and treatment of XXMD. The present study used a novel lipidomics approach to analyze the therapeutic effects of the active XXMD fraction on CCI. Besides, an exploratory correlation analysis was conducted between the effective constituents of the active XXMD fraction and the potential lipid biomarkers, making a bridge between the exogenous treatment and endogenous metabolism. Thus, two active compounds, cimifugin (H1) and 5-*O*-methylvisamminol, which can cross BBB, should be a special focus in future studies. We also built a network with a combination of the base material and lipid metabolites, which showed that the therapeutic effects of XXMD were closely correlated with its ability to regulate lipid metabolism. These results provide new evidence of the efficacy and mechanisms of the action of the constituents in XXMD. XXMD, a traditional Chinese medicine, exerted its pharmacological effects through a multi-component, multi-target mechanism. This methodology can be used in the research and development of novel active compounds and their metabolites of TCM.

## Author Contributions

JZ designed the research. ZJ conducted the experiments, analyzed the data, and drafted the manuscript. CWa helped with the performance of animal model and sample preparation. CWu helped to conduct the animal model. CT helped with the data analysis. All authors approved the final manuscript.

## Conflict of Interest Statement

The authors declare that the research was conducted in the absence of any commercial or financial relationships that could be construed as a potential conflict of interest.
