# Supplementary material for: Perturbed Lipidomic Profiles in Rats With Chronic Cerebral Ischemia Are Regulated by Xiao-Xu-Ming Decoction
Source: Front Pharmacol. 2019 Mar 19;10:264. doi: 10.3389/fphar.2019.00264 (PMC6433774; doi:10.3389/fphar.2019.00264)
Supplement: Supplementary file 1 [file Data_Sheet_1.pdf]

Perturbed Lipidomic Profiles in Rats with Chronic Cerebral Ischemia Are Regulated by Xiao-Xu-Ming Decoction

Zhixin Jia [a], Cai Tie [a], Caihong Wang [a], Caisheng Wu [a], Jinlan Zhang [a,*]

[a] *State Key Laboratory of Bioactive Substance and Function of Natural Medicines, Institute of Materia Medica, Chinese Academy of Medical Sciences & Peking Union Medical College, No. 2 Nanwei Road, Xicheng District, Beijing 100050, China.*

[*] Corresponding author. Phone: +8610-83154880 Fax: +8610-83154880

E-mail address: jessiejzx@163.com (Z. Jia), tiecai@imm.ac.cn (C. Tie), wangch@imm.ac.cn (C. Wang), wucsh@imm.ac.cn (C. Wu), zhjl@imm.ac.cn (J. Zhang).

S1 Text: The list of lipid standards

Sphingolipids: Sph(d17: 1), Sph(d18: 1), Sph(d17: 1)-1-P, Sph(d18: 1)-1-P, Cer(d18: 1/2: 0), Cer(d18: 1/2: 0)-1-P, Cer(d18: 1/4: 0), Cer(d18: 1/6: 0), Cer(d18: 1/8: 0), Cer(d18: 1/8: 0)-1-P, Cer(d18: 1/10: 0), Cer(d18: 1/12: 0), Cer(d18: 1/12: 0)-1-P, Cer(d18: 1/14: 0), Cer(d18: 1/16: 0), Cer(d18: 1/18: 1), Cer(d17: 1/18: 0), Cer(d18: 1/18: 0), Cer(d18: 1/16: 0)-1-P, Cer(d18: 1/18: 1)-1-P, Cer(d18: 1/20: 0), Cer(d17: 1/24: 1), Cer(d18: 1/22: 0), Cer(d18: 1/24: 1), Cer(d18: 1/24: 0), HexSph(d18: 1), dhSph(d17: 0), dhSph(d18: 0), dhS1P(d17: 1), dhS1P(d18: 1), dhCer(d18: 0/2: 0), GlcCer(d18: 1/8: 0), dhCer(d18: 0/6: 0), dhCer(d18: 0/8: 0), HexCer(d18: 1/12: 0), HexCer(d18: 1/16: 0), Hex-Cer(d18: 1/18: 1), HexCer(d18: 1/18: 0), dhCer(d18: 0/16: 0), Cer(d17: 1/18: 0), dhCer(d18: 0/18: 1),dhCer(d18: 0/18: 0), HexCer(d18: 1/24: 1), Cer(d17: 1/24: 1), dhCer(d18: 0/24: 1), dhCer(d18: 0/24: 0). Other kinds of lipids: PS(16: 0/18: 1), PG(16: 0/18: 1), PC(10: 0), PE(16: 0/18: 1), PC(17: 0/20: 4), LPC(17: 1/0: 0), PS(17: 0/20: 4), PE(17: 0/20: 4), PI(17: 0/20: 4), d5-DG ISTD MixⅡ, d5-TG ISTD MixⅠ, SM17: 0, PC (18: 0/18: 0), DG(18: 0/18: 0), PS(12: 0/12: 0), PE(12: 0/12: 0).

All the lipid standards were bought from Avanti (Alabaster, AL, USA).

S2 Text: Quantification of lipids

2.1 Preparation of standard solution

2.1.1 Sphingolipids

a) Preparation of blank matrix

Exact 100 mg bovine serum albumin (BSA) was dissolved in normal saline in 10 mL volumetric flask. The solution was called blank matrix.

b) Preparation of internal standards

1). *For plasma analysis:* Mixed standard solution was prepared at a concentration of 1000 pmol/mL, marked for IS-P. 2). *For brain tissue analysis*: The mixed standards mentioned in 1) can be detected in brain tissue. Then, exogenous compounds should be used as internal standards. Derived fatty acids, which were kindly provided by Professor Cai Tie in our research group, were selected, marked as IS-B with a concentration of 2000 pmol/mL. The names of each standard were listed in Table S6.

c) Preparation of standard solutions

The stock solution was prepared at a concentration of 1000 pmol/mL. The names of standards were listed in Table S6. Proper amount of stock solution was added to make solutions with concentrations of 25, 50, 100, 200, 500, 1000, 2000 and 4000 pmol/mL separately.100 μL blank matrixes was added into glass tube, and then, mixed standard solution with different concentrations were added, too. 20 μL internal standards were added into it, then with 1.5 mL methanol and 5 m MTBE. The mixture was vortexed for 15 min, and then 1.5 mL deionized water was added with 10 min centrifugation at a speed of 4500 rpm for phase separation. The upper organic phase was transformed into another glass tube and the lower aqueous phase was extracted again. 2 mL second-exaction-liquid was added into the aqueous phase, vortex for 15 min, then with centrifugation at a speed of 4500 rpm for 10 min. The upper organic phase was combined and dried under nitrogen. 100 μL methanol (with 0.1% FA, 1 mM ammonium formate) was used to dissolve.

d) Preparation of quality control (QC) samples

The solutions in “Preparation of standard solutions” with the concentration of 100,500, 2000 pmol/mL were the QC samples.

2.1.2 Other kinds of lipids

a) Second-extraction-liquid

322.6mL MTBE, 96.8mL methanol and 80.6 mL water was added into separating funnel. After extraction, the mixture was placed under room temperature for 4 hours. Then the upper layer was the second-extraction-liquid.

b) Preparation of internal standard for extraction

Proper amount of lipids standards (listed in Table S6) were weighted and added into 2 mL volumetric flask and diluted with methanol. 2 mL of this solution was transferred to another 10 mL volumetric flask and diluted with methanol which contained 0.005% BHT. Then this solution was the internal standard for extraction, marked IS-E.

c) Preparation of internal standard for quantification

Proper amount of lipids standards (listed in Table S6) were weighted and added into 5 mL volumetric flask and diluted with chloroform which contained 0.005% BHT. This solution was the internal standard for quantification, marked IS-Q.

d) Preparation of quality control (QC) samples

The mixed lipid standards solution with the concentration of 1 nmol/mL was the QC samples.

2.2 The extraction of lipids

The extraction was carried out by MTBE-MEOH-WATER three phase extract method (1).

100 μL samples (plasma or brain tissue) was added into glass tube, and then 100 μL IS-E, 20 μL internal standards for sphingolipids (IS-P for plasma and IS-B for brain tissue), 1.5 mL methanol and 5 mL MTBE was also added. The mixture was vortexed for 15 min, and then 1.5 mL deionized water was added to lead phase separation. After centrifugation at a speed of 4500 rpm for 10 min, the upper organic phase was transferred into another glass tube. 2 mL second-extraction-liquid was added into the lower aqueous phase was. After vortex for 5 min and centrifugation at a speed of 4500 rpm for 10min, the upper organic phase was combined with that of the first time. 2 mL mixed liquid was dried under nitrogen, and then redissolved with 20 μL methanol containing 0.1% FA and 1 mM ammonium formate. This part was for spingolipids analysis. The rest part was dried under nitrogen, and then redissolved with 80 μL IS-Q. This part was for analysis of other lipids.

2.3 Analysis with HPLC-MS/MS

2.3.1 HPLC coupled with triple quadrupole MS

Chromatographic conditions: Column: Peeke C8SR (150×3.0 mm, 3 μm). Mobile phase: A water (containing 0.1%FA, 1 mM ammonium formate); B methanol (containing 0.1% FA, 1 mM ammonium format). Flow rate: 0.5mL/min. Column temperature: 40℃.Gradient elution as following: 0-10 min, 80-100% B; 10-18 min, 100% B; 18-18.1min, 100-80% B; 18.1-25 min, 80% B.

Mass spectrometry conditions: Gas Temp: 350℃; Gas Flow: 10 L/min; Nebulizer: 30 psi; Capillary: 4000 V. This method contained 6segments: 0-1.5 min, segment 1, to waste; 1.5-7 min, segment 2, to MS; 7-12 min, segment 3, to MS; 12-14 min, segment 4, to MS; 14-16 min,

segment 5, to MS; 16-25 min, segment 6, to MS. The compounds eluted around the boundary point (± 0.5 min) of each segment were included in both segments. So, all compounds could be detected even if the retention time drifted. The details were listed in Table S3.

2.3.2 HPLC coupled with FTICRMS

Chromatographic conditions: Column: Waters Xterra MS C8 (2.1×100mm, 3.5μm). Mobile phase: C, acetonitrile: isopropanol= 5: 2, *v/v* (containing 2mM ammonium acetate, 0.1% formic acid); D, 2 mM ammonium acetate, 0.1% formic acid aqueous solution. Gradient elution as following, 0-1 min 10%A, 1-2 min 10-30%A, 2-4 min 30-50%A, 4-8 min 50-70%A, 8-12 min 70-100%A, 12-24 min 100%A, 24-24.5 min 100-10%A, 24.5-30 min 10%A. Flow rate: 0.35mL/min. Column temperature: 40℃.Injection volume: 10μL. Injection temperature: 4℃. Needle washing: 3000 μL methanol.

MS conditions: Ion source: ESI; Detection mode: positive; ion spray voltage: 4.5kV; sheath gas flow rate(N2): 50arb; auxiliary gas flow rate (N2): 20arb; sweep gas flow rate (N2): 3arb; capillary voltage: 35V; capillary temperature: 275℃; Scan mode: FT Full Scan; range: m/z 50-1200 Da; resolution100, 000.

S3 Text: Metabolites identification after oral administration of XXMD

3.1 Sample preparation

Plasma: There were 3 samples in each time point (0.5h, 2.5h and 8h after oral administration) which were combined before protein precipitation. 2 mL mixture was taken into 15 mL centrifuge tube and 10 mL methanol was used to precipitate protein. After vortex for 30 s and centrifugation at a speed of 1721g for 10 min, the supernatant was dried under nitrogen and redissolved in 100

μL methanol. The solution mentioned above was then centrifuged at a speed of 15493 g for 10 min and analyzed.

Brain tissue: 1 mL homogenate of the brain tissue was taken into 15 mL centrifuge tube and 5 mL methanol was used to precipitate protein. The remaining steps were the same as plasma samples.

3.2 HPLC coupled with MS/MS analysis

Chromatographic conditions: Colum: Thermo BDS HYPERSIL C18 column (150×2.1 mm, 3 mm). Mobile phase: aqueous solution, containing 0.1% formic acid (A); acetonitrile (B). Gradient elution as following: 0-5 min, A: 95-95%, B: 5-5%; 5-25 min, A: 95-70%, B: 5-30%; 25-35 min, A: 70-60%, B: 30-40%; 35-45 min, A: 60-20%, B: 40-80%; 45-50 min, A: 20-20%, B: 80-80%; 50-51 min, A: 20-95%, B: 80-5%; 51-60 min, A: 95%-95%, B: 5%-5%. Injection volume: 5 μL; Column temperature: 30℃; Flow rate: 0.2mL/min.

Mass spectrometry conditions: Ion source: ESI; Detection mode: positive; ion spray voltage: 4.0kV; sheath gas flow rate(N2): 40arb; auxiliary gas flow rate (N2) : 10arb; sweep gas flow rate (N2) : 3arb; capillary voltage: 40V; capillary temperature: 250℃. Mass spectra data were recorded by full-scan mass analysis from *m/z* 100 to 1500 at a resolving power of 50,000 with data-dependent MS^2 analysis triggered by the two most abundant ions from full-scan mass analysis, followed by MS^3 analysis of the most abundant ions of MS^2. A repeat count of one prior to exclusion was utilized for dynamic exclusion, and the repeat duration was 10 seconds. The time of mass-to-charge value residing on the exclusion list (the list size was set to 50) was 30 s after the data-dependent MS^2 experiment. Collision-induced dissociation (CID) was conducted

with an isolation width of 1 Da. Xcalibur software (Version 2.0; Thermo Scientific) was used for data acquisition and reduction.

S4 Text: The establishment and optimization of lipids quantification method

4.1 HPLC coupled with triple quadrupole MS

In our previous study, the HPLC coupled with triple quadrupole MS methods for sphingolipids quantification have been established. 43 spingolipids could be detected and quantified via these methods (2). In this study, these methods were optimized on the following two aspects: 1) Analysis time. Each sample should be analyzed for three times in the previous methods which consumed about 1 hour. So, we expected to restrict the analysis time into 30 minutes. 2) Detected compounds. Sphingomyelin (SM) had high abundance in biological samples, which could be detected via the FTICRMS method (3). Thus, this method could get rid of SM and take Sph, Cer, dhCer, Cer-1P, dhCer-1P and glucoceramide into consideration only. As a result, only one analysis cycle was needed. Besides, the former methods only contained compounds with standard substance, except two ion pairs. This time, we add more ion pairs with no standard substance to enrich the method which made the detection for biological samples more comprehensive.

To achieve good separation and profile the chemical components, different columns and elution programs were investigated. Such as, shiseido capcell pak C8 column, capcell pak C1 column, Waters C8 Sunfire column, Agilent CE C8 column, and Peeke C8SR column. Peeke C8SR showed the best separation efficiency. Different mobile phases and elution programs were also tested. Water (containing 0.1% FA, 1 mM ammonium format) and methanol (containing

0.1% FA, 1 mM ammonium format) was chosen for analysis. Finally, an optimized method for sphingolipids was established as described in “2.3.1”.

4.2 HPLC coupled with FTICRMS

For the other kinds of lipids, the established HPLC coupled with FTICRMS method in our previous study could detect 44 compounds in one analyze cycle in one hour. These compounds covered diacylglycerol (DG), triacylglycerol (TG), phosphatidylcholine (PC) and phosphatidylethanolamine (PE) (4). The optimization of this method was limiting the analysis time into 30 minutes. The lipid data analyzer (LDA) was used to do the quantification and its library was expanded this time. The chromatography and MS conditions were also optimized. The analysis sequence was improved with the addition of needle washing program after each sample program. So that sample residual on the injection needle and needle blocking were avoided efficiently.

These two methods described above constructed the platform for lipids quantification (Fig. S2).

Table S1 Detailed information of 12 herbs in XXMD

Chinese name	English name	Latin name	Family	Used part
Fang feng	Divaricate saposhnikovia root	Radix Saposhnikoviae	Saposhnikovia divaricata (Turcz.) Schischk.	Root
Huang qin	Baical skullcap root	Radix Scutellariae	Scutellaria baicalensis Georgi.	Root
Shao yao	Debark peony root	Radix Paeoniae Alba	Paeonia lactiflora Pall.	Root
Gan cao	Liquorice root	Radix Glycyrrhizae	Glycyrrhiza uralensis Fisch.	Root
Sheng jiang	Fresh ginger	Rhizoma Zingiberis Recens	Zingiber officinale Rosc.	Root
Fang ji	Mealy fangji root	Radix Stephaniae Tetrandrae	Stephania tetrandra S. Moore.	Root
Ren shen	Ginseng	Radix Ginseng	Panax ginseng C. A. Mey.	Root
Gui zhi	Cassia twig	Ramulus Cinnamomi	Cinnamomum cassia Presl.	Twig
Xing ren	Bitter apricot seed	Semen Armeniacae Amarum	Prunus armeniaca L. var. ansu Maxim.	Seed
Ma huang	Ephedra	Herba Ephedrae	Ephedra sinica Staph.	Stem
Chuan xiong	Sichuan lovage rhizome	Rhizoma Ligustici Chuanxiong	Ligusticum chuanxiong Hort.	Root
Fu zi	Prepared common monk shood branched root	Radix Aconiti Lateralis Preparata	Aconitum carmichaeli Debx.	Root

Table S2 Content levels of main component in the active fraction of XXMD (5)

Compounds	Content (%)	Compounds	Content (%)
Ephedrine	0.107	Wogonoside	2.302
Pseudoephedrine	0.297	Baicalein	1.458
Paeoniflorin	0.395	Liquiritingenin	0.438
Prim-O-glucosylcimifugin	1.207	Cinnamic acid	0.571
Cimicifugin	0.408	Ferulic acid	0.511
5-O-methylvisamminoside	0.367	Chrysin	0.355
Baicalin	5.102	Oroxylin A	0.215
Liquiritin	0.220	Wogonin	0.369
Isoliquiritin	0.394	Glycyrrhizic acid	0.600
Oroxylin A-7-O-glucuronide	1.932		

Table S3 Segments and ion pairs

Segments	2				
Compound Name	Precursor Ion	Product Ion	Dwell	Fragmentor	Collision Energy
HexSph 18:1	462.1	282.2	60	130	21
dhSph 18:0-1P	382.1	284.2	60	80	20
Sph 18:1-1P	380.3	264.1	60	70	15
Sph 17:1-1P	366.1	250	60	70	15
dhSph 18:0	302.1	284.2	60	120	5
Sph 18:1	300.3	282.3	60	90	5
Sph 17:1	286.1	268.1	60	100	7
Segments	3				
Compound Name	Precursor Ion	Product Ion	Dwell	Fragmentor	Collision Energy
HexCer 16:1	698.2	264.2	45	140	36
HexCer 14:0	672.2	264.1	45	150	36
HexCer 14:1	670.2	264.1	45	150	36
HexCer 12:0	644.2	264.1	45	150	36
HexCer 12:1	642.2	264.1	45	150	36
HexCer 8:0	588.2	264.2	45	100	36
Cer 8:0-1P	506.1	264.2	45	80	25
Cer 12:0	482.4	264.1	45	140	20

Cer 10:0	454.2	264.1	45	140	15
dhCer 8:0	428.2	266.2	45	150	25
Segments	4				
Compound Name	Precursor Ion	Product Ion	Dwell	Fragmentor	Collision Energy
HexCer 22:1	782.3	264.2	15	155	39
HexCer 20:0	756.3	264.2	15	155	39
HexCer 20:1	754.3	264.2	15	150	39
HexCer 18:0	728.2	264.2	15	150	39
HexCer 18:1	726.1	264.2	15	160	36
HexCer 16:0	700.2	264.2	15	140	36
HexCer 16:1	698.2	264.2	15	140	36
HexCer 14:0	672.2	264.1	15	150	36
HexCer 14:1	670.2	264.1	15	150	36
Cer 16:0-1P	618.1	264.1	15	110	30
Cer 16:1-1P	616.1	264.1	15	110	30
dhCer 20:0	596.3	266.2	15	145	30
Cer 14:0-1P	590.2	264.1	15	105	25
Cer 14:1-1P	588.2	264.1	15	105	25
dhCer 18:0	568.2	266.2	15	140	25
Cer 18:0	566.4	264.2	15	150	25

dhCer 18:1	566.2	266.2	15	140	30
Cer 18:1	564.4	264.1	15	130	20
Cer 12:0-1P	562.1	264.1	15	100	26
Cer 12:1-1P	560.1	264.1	15	100	26
Cer (17:1) 18:0	552.4	250.1	15	130	30
dhCer 16:0	540.2	266.2	15	160	30
Cer 16:0	538.4	264.2	15	140	20
Cer 14:0	510.4	264.1	15	120	20
Cer 12:0	482.4	264.1	15	140	20
Segments	5				
Compound Name	Precursor Ion	Product Ion	Dwell	Fragmentor	Collision Energy
HexCer 24:0	812.2	264.1	11	160	36
HexCer 24:1	810.2	264.1	11	160	36
HexCer 22:0	784.3	264.2	11	160	39
HexCer 22:1	782.3	264.2	11	155	39
HexCer 20:0	756.3	264.2	11	155	39
HexCer 20:1	754.3	264.2	11	150	39
Cer 22:1-1P	700.2	264.1	11	125	35
Cer 26:1	676.5	264.1	11	140	35
dhCer 20:1-1P	674.2	266.2	11	120	25

Cer 20:0-1P	674.2	264.1	11	120	35
Cer 20:1-1P	672.2	264.1	11	120	35
dhCer 24:0	652.3	266.2	11	180	30
Cer 24:0	650.4	264.1	11	140	30
dhCer 24:1	650.3	266.2	11	150	35
Cer 24:1	648.3	264.1	11	140	30
dhCer 18:0-1P	648.2	266.2	11	120	25
dhCer 18:1-1P	646.2	266.2	11	120	25
Cer 18:0-1P	646.2	264.1	11	120	35
Cer 18:1-1P	644.2	264.1	11	120	35
Cer (17:1) 24:1	634.5	250.1	11	140	10
dhCer 22:0	624.3	266.1	11	140	30
Cer 22:0	622.3	264.1	11	140	25
dhCer 16:0-1P	620.2	266.2	11	115	25
dhCer 16:1-1P	618.2	266.2	11	110	25
Cer 16:0-1P	618.1	264.1	11	110	30
Cer 16:1-1P	616.1	264.1	11	110	30
dhCer 20:0	596.3	266.2	11	145	30
Cer 20:0	594.4	264.1	11	140	25
Cer 14:0-1P	590.2	264.1	11	105	25

dhCer 18:0	568.2	266.2	11	140	25
Cer 18:0	566.4	264.2	11	150	25
dhCer 18:1	566.2	266.2	11	140	30
Cer (17:1) 18:0	552.4	250.1	11	130	30
Segments	6				
Compound Name	Precursor Ion	Product Ion	Dwell	Fragmentor	Collision Energy
dhCer 28:0-1P	788.3	266.2	15	135	36
Cer 28:0-1P	786.4	264.1	15	135	35
dhCer 28:1-1P	786.3	266.2	15	145	35
Cer 28:1-1P	784.4	264.1	15	125	35
dhCer 26:0-1P	760.2	266.2	15	125	35
Cer 26:0-1P	758.4	264.1	15	125	35
dhCer 26:1-1P	758.2	266.2	15	125	35
Cer 26:1-1P	756.1	264.1	15	125	35
dhCer 24:0-1P	732.2	266.2	15	125	25
Cer 24:0-1P	730.3	264.1	15	125	35
dhCer 24:1-1P	730.2	266.2	15	125	25
Cer 24:1-1P	728.2	264.1	15	125	35
dhCer 22:0-1P	704.2	266.2	15	125	25
Cer 22:0-1P	702.3	264.1	15	130	35

dhCer 22:1-1P	702.2	266.2	15	125	25
Cer 22:1-1P	700.3	264.1	15	130	32
Cer 26:0	678.5	264.1	15	140	35
Cer 26:1	676.5	264.1	15	140	35
dhCer 20:0-1P	676.2	266.2	15	120	25
dhCer 20:1-1P	674.2	266.2	15	120	25
dhCer 24:0	652.3	266.2	15	180	30
Cer 24:0	650.4	264.1	15	140	30
dhCer 24:1	650.3	266.2	15	150	35
dhCer 18:0-1P	648.2	266.2	15	120	25
dhCer 18:1-1P	646.2	266.2	15	120	25
dhCer 16:0-1P	620.2	266.2	15	115	25

Table S4 Metabolites after oral administration

No.	RT (min)	M+H	Error	Proposed formula	MS^n	Name	Parent structure	Metabolism	No.
1	23.9	293.1018	-0.165	$C_{15}H_{17}O_6$	MS^2[293]: 275(100), 233(64);<br>MS^3[233]: 233(100)	Cimifugin-methyl	H1	- Methyl	M1
2	25.07	293.1018	-0.165	$C_{15}H_{17}O_6$	MS^2[293]: 275(100), 221(48);<br>MS^3[275]: 257(65), 221(100)	Cimifugin-methyl	H1	- Methyl	M2
3 [a]	20.03	307.1174	-0.215	$C_{16}H_{19}O_6$	MS^2[307]: 289(83), 235(100);<br>MS^3[235]: 207(100)	Cimifugin	H1	-	H1
4	16.08	321.0964	-0.48	$C_{16}H_{17}O_7$	MS^2[321]: 303(92), 273(22), 249(100);<br>MS^3[249]: 231(55), 221(100)	Cimifugin+O-2H	H1	+O-2H	M3
5	17.48	323.1128	0.27	$C_{16}H_{19}O_7$	MS^2[323]: 247(100);<br>MS^3[247]: 232(100)	Cimifugin+O	H1	+O	M4

6	17.46	469.1698	-0.693	$C_{22}H_{29}O_{11}$	MS^2[469]: 307(100);	Cimifugin+glucosyl	H1	+Glu	M5
7	17.61	483.149	-0.703	$C_{22}H_{27}O_{12}$	MS^3[307]: 289(88), 235(100)	Cimifugin+glucuronidation	H1	+GluA	M6
					MS^2[483]: 307(100);				
					MS^3[307]: 289(85), 235(100)				
8	33.5	257.0809	0.064	$C_{15}H_{13}O_4$	MS^2[257]: 239(97), 211(34), 147(100), 137(83);	Liquiritigenin	H2	-	H2
					MS^3[147]: 119(100)				
9	24.43	419.1339	0.241	$C_{21}H_{23}O_9$	MS^2[419]: 257(100);	Liquiritigenin+glucosyl	H2	+Glu	M9
					MS^3[257]: 239(100), 147(99)				
10	19.23	433.1128	-0.224	$C_{21}H_{21}O_{10}$	MS^2[433]: 257(100);	Liquiritigenin+glucuronidation	H2	+GluA	M10
					MS^3[257]: 239(100), 147(99), 137(87)				
11	25.35	433.1128	-0.224	$C_{21}H_{21}O_{10}$	MS^2[433]: 257(100);	Liquiritigenin+glucuronidation	H2	+GluA	M11
					MS^3[257]: 239(88), 147(89), 137(100)				
12	27.41	431.0983	0.848	$C_{21}H_{19}O_{10}$	MS^2[431]: 255(100);	Chrysin+glucuronidation	H3	+GluA	M12
					MS^3[255]: 255(100), 237(90), 153(55)				
13	19.9	549.1598	-0.468	$C_{26}H_{29}O_{13}$	MS^2[549]: 531(100), 411(81);	Chrysin+arabinose+	H3	+Ara	M13

					MS^3[531]: 513(100), 411(44)	glucosyl		+Glu	
14	26.8	447.0918	-0.388	$C_{21}H_{19}O_{11}$	MS^2[447]: 271(100);	Baicalein+glucuronidation	H4	+GluA	M14
15	28.03	447.0918	-0.388	$C_{21}H_{19}O_{11}$	MS^3[271]: 271(100), 253(22)	Baicalein+glucuronidation	H4	+GluA	M15
					MS^2[447]: 411(100), 271(60);				
					MS^3[271]: 271(50), 253(100), 169(20)				
16	21.75	609.1434	-1.612	$C_{27}H_{29}O_{16}$	MS^2[609]: 447(100);	Baicalein+glucosyl+	H4	+Glu	M16
					MS^3[447]: 271(100)	glucuronidation		+GluA	
17	21.68	623.1234	-0.876	$C_{27}H_{27}O_{17}$	MS^2[623]: 447(100);	Baicalein+2 glucuronidation	H4	+2 GluA	M17
					MS^3[447]: 271(100)				
18	28.43	461.1079	0.062	$C_{22}H_{21}O_{11}$	MS^2[461]: 285(100);	Wogonin+glucuronidation	H5	+GluA	M18
					MS^3[285]: 270(100)				
19	22.17	637.1395	-0.427	$C_{28}H_{29}O_{17}$	MS^2[637]: 285(100);	Wogonin+2 glucuronidation	H5	+2 GluA	M19
					MS^3[285]: 270(100)				
20 [a]	25.52	291.1231	0.399	$C_{16}H_{19}O_5$	MS^2[291]: 219(100)	5-O-methylvisamminol	H6	- Glu	M20
21	33.7	277.1074	0.35	$C_{15}H_{17}O_5$	MS^2[277]: 259(100);	Hamaudol	H7	-	H7
					MS^3[259]: 217(100), 205(95)				

22	26.86	439.1602	0.326	$C_{21}H_{27}O_{10}$	MS^2[439]: 277(100);	Hamaudol+glucosyl	H7	+Glu	M21
23	27.25	461.1079	0.062	$C_{22}H_{21}O_{11}$	MS^3[277]: 259(100), 205(55)<br>MS^2[461]: 285(100);<br>MS^3[285]: 270(100)	Oroxyloside	H8	-	H8
24	38.71	315.0864	0.085	$C_{17}H_{15}O_6$	MS^2[315]: 300(100), 282(62), 199(9);<br>MS^3[300]: 285(79), 282(100)	5,7-Dimethoxy-4',6-dihydroxy flavone	H9	-	H9
25	28.66	491.1186	0.197	$C_{23}H_{23}O_{12}$	MS^2[491]: 315(100);<br>MS^3[315]: 300(100), 285(18), 169(0.15)	5,7-Dimethoxy-4',6-dihydroxy flavone + Glucuronidation	H9	+GluA	M22
26	47.31	471.3479	1.013	$C_{30}H_{47}O_4$	MS^2[471]: 453(60), 435(26), 425(100);<br>MS^3[425]: 407(100)	Enoxolone	H10	-	H10
27	24.69	507.1138	0.582	$C_{23}H_{23}O_{13}$	MS^2[507]: 331(100);<br>MS^3[331]: 316(100), 183(9), 169(51)	Jaceosidin+glucuronidation	H11	- Glu<br>+GluA	M7
28	25.09	477.102	-0.753	$C_{22}H_{21}O_{12}$	MS^2[477]: 301(100);<br>MS^3[301]: 286(100)	Hispidulin+glucuronidation	H12	+GluA	M8

[a] Metabolites detected in both the brain tissue and plasm

Table S5 Potential biomarker and their change tendency

Plasma				Brain tissue			
Model vs Sham operation		Model vs Model+XXMD		Model vs Sham operation		Model vs Model+XXMD	
38		73		52		100	
Name	tendency	Name	tendency	Name	tendency	Name	tendency
dhCer24: 0	↑	Cer16: 0-1P	↑	Cer24: 1	↑	Cer18: 0-1P	↑
HexCer8: 0	↑	Cer24: 0	↓	LPC 16: 0	↓	Cer18: 1	↑
Sph18: 1	↑	DG 34: 2	↓	LPC 18: 2	↓	Cer22: 0-1P	↓
LPC 14: 0	↓	DG 44: 12	↓	LPC 18: 3	↓	Cer22: 1-1P	↓
LPC 16: 1	↓	LPC 16: 0	↓	LPC 22: 6	↓	Cer24: 0-1P	↓
LPC 18: 3	↓	LPC 16: 1	↓	LPE 16: 0	↓	Cer24: 1	↑
LPC 18O: 0	↑	LPC 18: 0	↓	LPE 18: 1	↓	Cer24: 1-1P	↓
LPC 20: 3	↓	LPC 18: 1	↓	LPE 20: 3	↓	Cer26: 0-1P	↓
LPC 20: 5	↓	LPC 18: 2	↓	LPE 20: 4	↓	dhCer16: 0-1P	↓
LPC 22: 5	↓	LPC 18: 3	↓	LPE 20O: 0	↓	dhCer18: 0-1P	↓
LPE 18: 1	↓	LPC 20: 0	↓	PC 32: 0	↓	dhCer24: 0-1P	↓
PC 32: 0	↓	LPC 20: 4	↓	PC 34: 0	↓	dhCer24: 1-1P	↓
PC 34: 3	↓	LPC 20: 5	↓	PC 36: 0	↓	dhCer24: 1	↑
PC 36: 1	↓	LPC 22: 6	↓	PC 36: 5	↓	dhCer26: 1-1P	↓
PC 36: 3	↓	LPE 16: 0	↓	PC 38: 6	↓	Sph18: 1-1P	↓
PC 38: 5	↓	LPE 18: 1	↑	PC 40: 4	↓	Sph18: 1	↓
PC 38: 6	↓	PC 36: 5	↓	PC 40: 6	↓	DG 38: 4	↓
PC 38: 8	↓	PC 38: 2	↓	PC 40: 8	↓	DG 40: 10	↓
PC 40: 10	↓	PC 40: 8	↓	PE 22: 0	↓	DG 42: 10	↓
PC 40: 7	↓	PE 22: 0	↑	PE 35: 1	↓	DG 44: 10	↓
PE 22: 0	↓	PE 39: 6	↑	PE 35: 2	↓	DG 44: 11	↓
PE 35: 1	↓	PE 40: 4	↑	PE 36O: 3	↓	LPC 22: 6	↑
PE 35: 2	↓	PE 40: 6	↑	PE 36P: 1	↓	LPE 16: 0	↑
PE 36: 2	↓	PE 41: 5	↑	PE 38: 4	↓	LPE 18: 1	↑
PE 36: 4	↓	PI 34: 2	↑	PE 38: 6	↓	LPE 20: 3	↑
PE 38: 1	↓	PI 34P: 3	↑	PE 38O: 3	↓	LPE 20: 4	↑
PE 39: 5	↓	PI 36: 5	↑	PE 39: 0	↓	LPE 22: 1	↑
PE 40: 6	↓	PI 40: 6	↑	PE 39: 5	↓	PC 34: 2	↓
PE 40O: 4	↓	PI 40: 7	↑	PE 40: 6	↓	PC 34: 4	↓

PE 41: 5	↓	PS 36P: 1	↑	PE 40: 7	↓	PC 36: 3	↓
SM 24d: 1	↓	SM 22: 0	↓	PE 40O: 5	↓	PC 36O: 2	↓
SM 24d: 4	↓	SM 24: 1	↓	PE 41: 5	↓	PC 36P: 3	↓
SM 26: 1	↓	SM 24d: 1	↓	PE 42P: 2	↓	PC 38: 1	↓
TG 56: 5	↓	SM 26: 1	↓	PI 40: 7	↓	PC 38O: 1	↓
TG 56: 6	↓	SM 26d: 1	↓	PS 38: 6	↓	PC 40: 5	↓
TG 56: 7	↓	TG 34P: 1	↓	TG 50: 2	↑	PE 22: 0	↑
TG 56: 8	↓	TG 52: 1	↓	TG 50: 3	↑	PE 31: 0	↑
TG 58: 8	↓	TG 52: 2	↓	TG 52: 4	↑	PE 34P: 1	↑
		TG 52: 3	↓	TG 52: 5	↑	PE 35: 1	↑
		TG 52: 4	↓	TG 54: 3	↑	PE 36: 0	↑
		TG 52P: 0	↓	TG 54: 4	↑	PE 36O: 3	↑
		TG 52P: 1	↓	TG 54: 5	↑	PE 36P: 1	↑
		TG 54: 0	↓	TG 54: 7	↑	PE 38: 4	↑
		TG 54: 1	↓	TG 56: 4	↑	PE 38: 6	↑
		TG 54: 2	↓	TG 56: 5	↑	PE 38O: 3	↑
		TG 54: 3	↓	TG 56: 6	↑	PE 38P: 4	↑
		TG 54: 4	↓	TG 56: 7	↑	PE 39: 5	↑
		TG 54: 5	↓	TG 58: 2	↑	PE 39: 6	↑
		TG 54: 6	↓	TG 58: 9	↑	PE 40: 4	↑
		TG 54P: 1	↓	TG 58P: 11	↑	PE 40: 6	↑
		TG 56: 0	↓	TG 60: 12	↑	PE 40: 7	↑
		TG 56: 1	↓	TG 60: 2	↑	PE 40P: 2	↑
		TG 56: 2	↓			PE 41: 5	↑
		TG 56: 3	↓			PE 42P: 2	↑
		TG 56: 4	↓			PI 38P: 5	↑
		TG 56: 5	↓			PI 40: 7	↑
		TG 56: 6	↓			PS 40: 6	↑
		TG 56: 7	↓			SM 16: 0	↓
		TG 56: 8	↓			SM 16: 1	↓
		TG 58: 0	↓			SM 18: 1	↓
		TG 58: 2	↓			SM 18d: 1	↓
		TG 58: 3	↓			SM 24: 0	↓
		TG 58: 4	↓			SM 24: 1	↓
		TG 58: 5	↓			SM 24d: 1	↓

TG 58: 6	↓
TG 60: 2	↓
TG 60: 3	↓
TG 60: 4	↓
TG 60: 5	↓
TG 62: 2	↓
TG 62: 3	↓
TG 62: 4	↓
TG 62: 7	↓

TG 46: 0	↓
TG 48: 0	↓
TG 48: 2	↓
TG 50: 1	↓
TG 50: 2	↓
TG 50: 3	↓
TG 52: 0	↓
TG 52: 1	↓
TG 52: 2	↓
TG 52: 3	↓
TG 52: 4	↓
TG 52: 5	↓
TG 52: 6	↓
TG 52P: 0	↓
TG 54: 0	↓
TG 54: 1	↓
TG 54: 2	↓
TG 54: 3	↓
TG 54: 4	↓
TG 54: 5	↓
TG 54: 6	↓
TG 54: 7	↓
TG 56: 1	↓
TG 56: 2	↓
TG 56: 4	↓
TG 56: 5	↓
TG 56: 6	↓
TG 56: 7	↓
TG 56: 8	↓
TG 58: 1	↓
TG 58: 2	↓
TG 58: 6	↓
TG 58: 9	↓
TG 58P: 11	↓
TG 60: 12	↓

Table S6 Standards and internal standards

QQQ method					FT method		
Standards			IS-P	IS-B	Standards (QC)	IS-E	IS-Q
HexSph 18: 1	Cer 10: 0	dhCer 18: 0	Sph 17: 1	Derivatized FA C6	PC(18: 0/18: 0)	PS(16: 0/18: 1)	d5-TG ISTD Mix Ⅰ
dhSph 17: 1	HexCer 12: 0	Cer 16: 0-1P	Sph 17: 1-1P	Derivatized FA C14	DG(18: 0/18: 0)	PG(16: 0/18: 1)	d5-DG ISTD Mix Ⅱ
Sph 18: 1	Cer 12: 0	Cer 18: 1-1P	dhCer 8: 0	Derivatized FA C22	PS(12: 0/12: 0)	PC(10: 0)	PC(17: 0/20: 4)
dhSph 18: 0	Cer 14: 0	Cer 20: 0	Cer 8: 0-1P	Derivatized FA C28	PE(12: 0/12: 0)	PE(16: 0/18: 1)	PE(17: 0/20: 4)
dhSph 17: 0-1P	HexCer 16: 0	HexCer 24: 1	Cer (17: 1) 18: 0				PS(17: 0/20: 4)
Sph 18: 1-1P	Cer 12: 0-1P	Cer 22: 0	Cer (17: 1) 24: 1				PI(17: 0/20: 4)
dhSph 18: 0-1P	HexCer 18: 1	Cer 24: 1					LPC(17: 1/0: 0)
Cer 4: 0	Cer 16: 0	dhCer 24: 1					
Cer 2: 0-1P	HexCer 18: 0	Cer 24: 0					
Cer 6: 0	Cer 18: 1	Cer 26: 1					
HexCer 8: 0	dhCer 16: 0	dhCer 24: 0					
dhCer 6: 0	dhCer 18: 1	Cer 26: 0					
Cer 8: 0	Cer 18: 0						

Figure S1. The identified compounds and their structures of XXMD by our previous work (6).

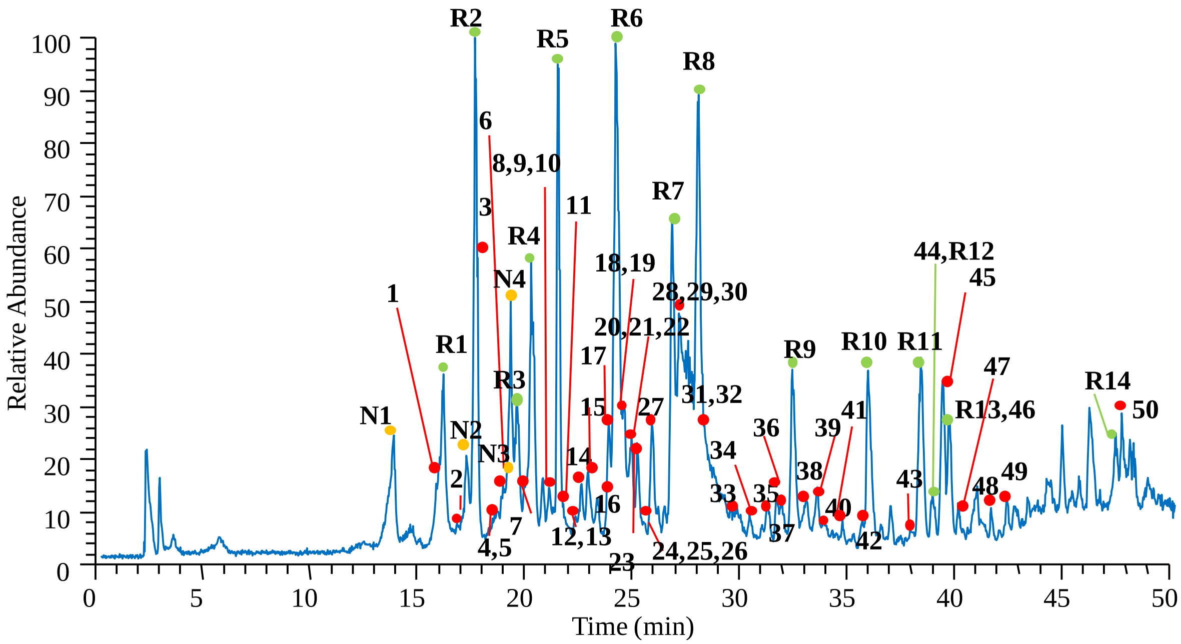

A 50 chemical components (red indication,**1-50**) of XXMD was discovered and identified based on the 14 templated compounds (green target recognition, **R1-R14**). These results showed that this technology has the advantage of rapidly identifying components of Chinese prescription. Notably, the unrelated peak is the compounds whose structures exhibited significant difference from the 14 templated compounds (such as orange indication, **N1-4**). (6)

Figure S2. The strategy for lipids analysis

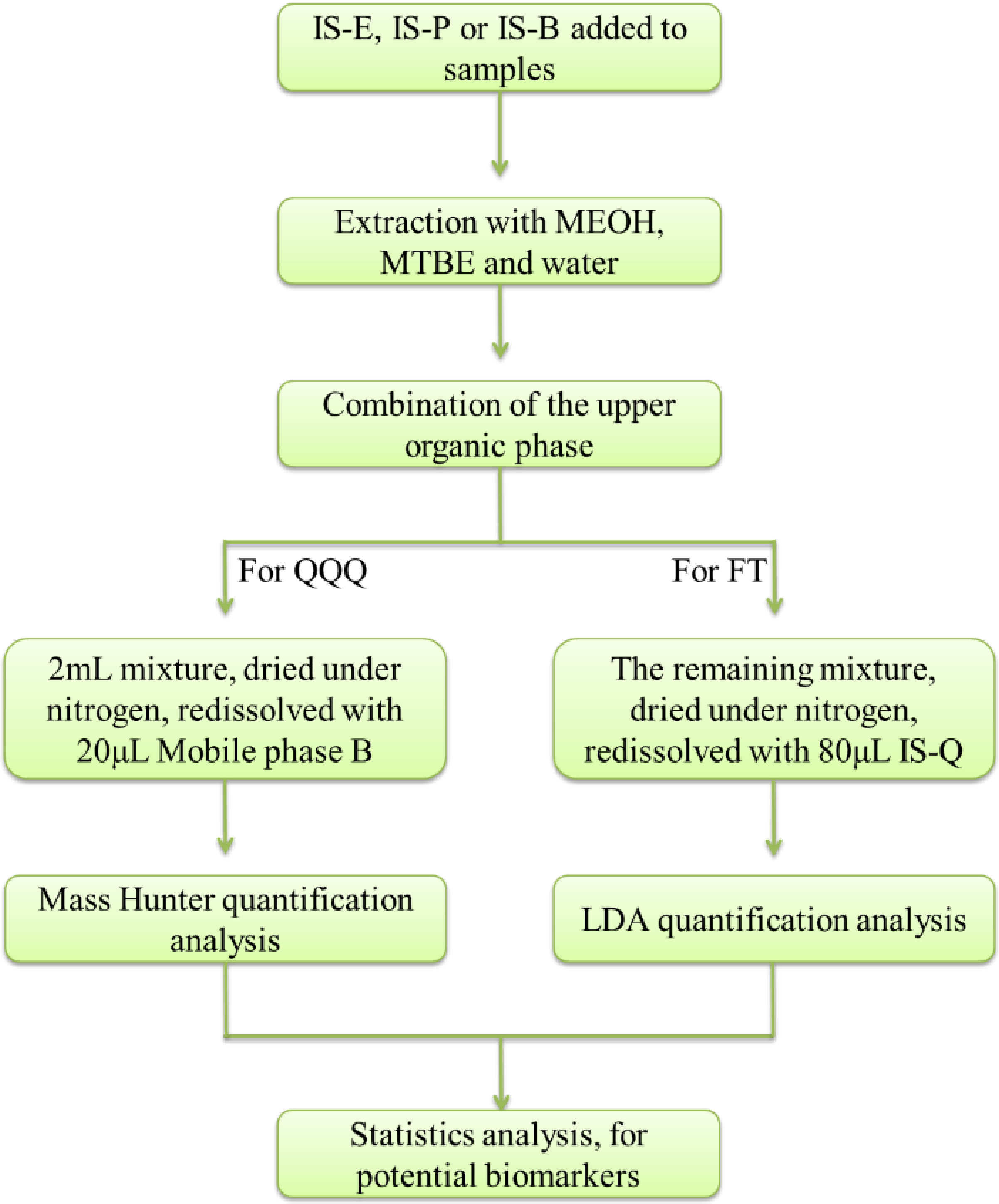

Figure S3. Light microscopic photographs of pathological section. The first column represented the sham operation group. The second column represented the model group. And the third column represented the model+XXMD group. The first row showed the pictures of Nissle's staining of hippocampal CA1 area which were magnified 200 times. Pictures in the second row were the Nissle's staining of cortex which was magnified 200 times. The third row showed pictures of white matter's KB staining which were magnified 100 times. The forth row were magnified 4 times of the third row.

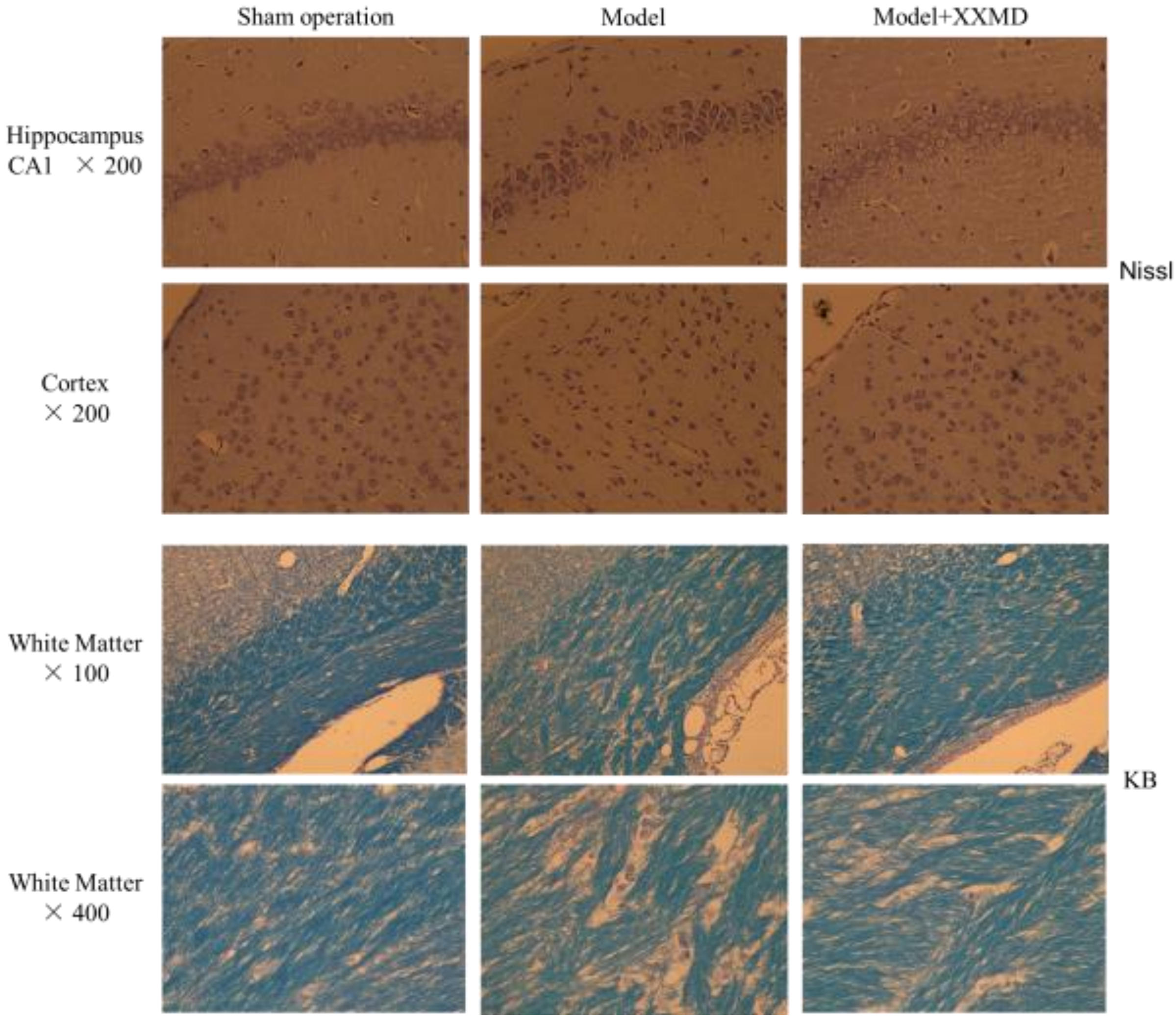

Figure S4. Bar graphs of compounds' concentration detected in plasma. (* stands for comparison with sham operation group, # stands for comparison with model group. Single symbol means P<0.05 while double means P<0.01).

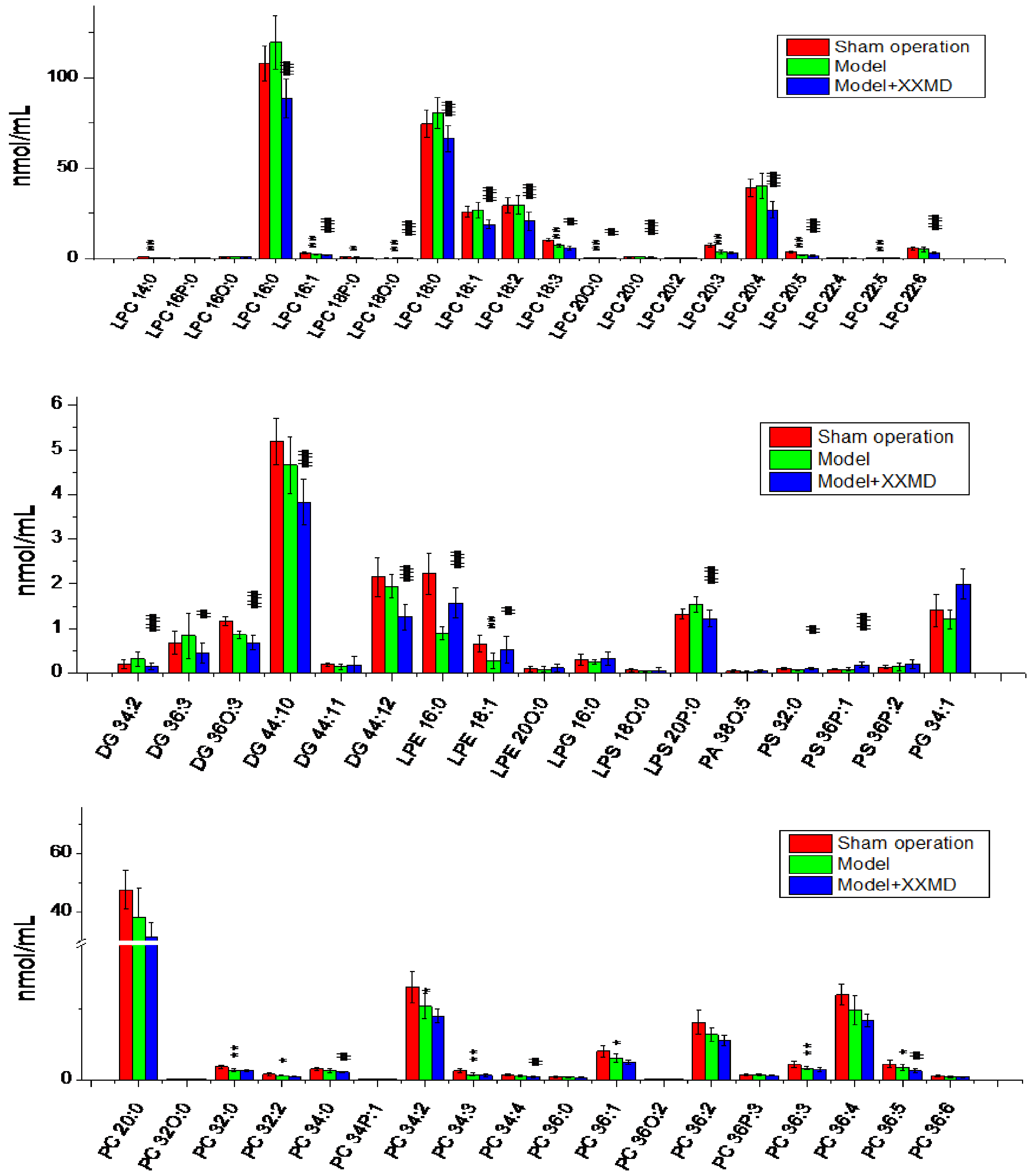

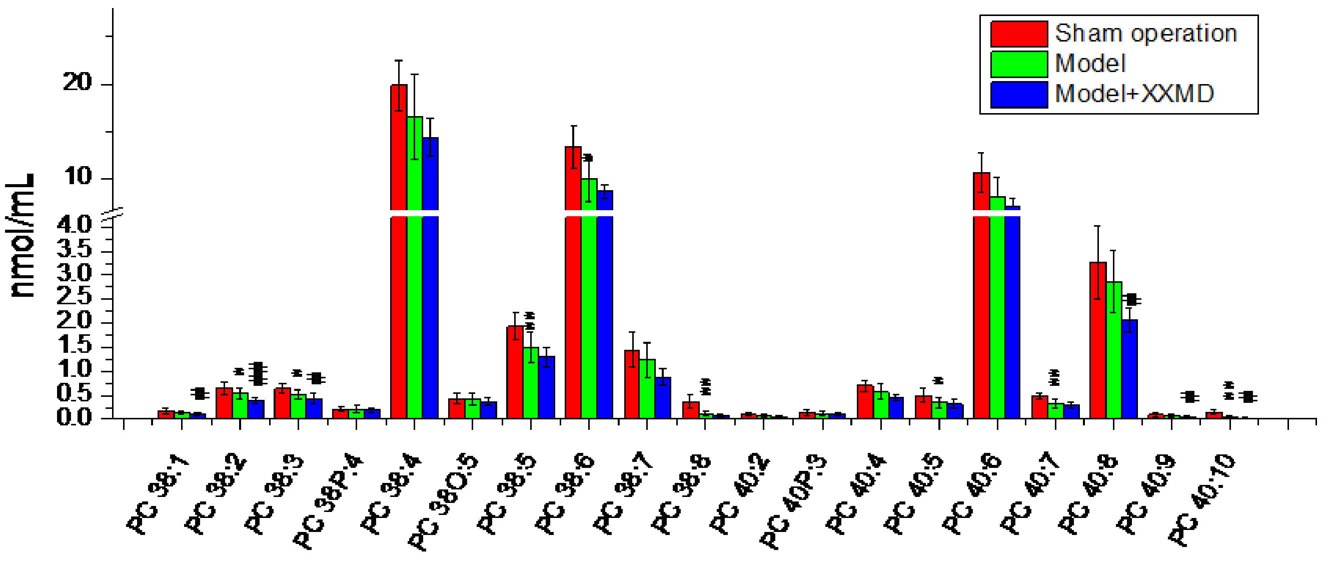
Sham operation
Model
Model+XXMD
nmol/mL
PC 38:1
PC 38:2
PC 38:3
PC 38P:4
PC 38:4
PC 38O:5
PC 38:5
PC 38:6
PC 38:7
PC 38:8
PC 40:2
PC 40P:3
PC 40:4
PC 40:5
PC 40:6
PC 40:7
PC 40:8
PC 40:9
PC 40:10

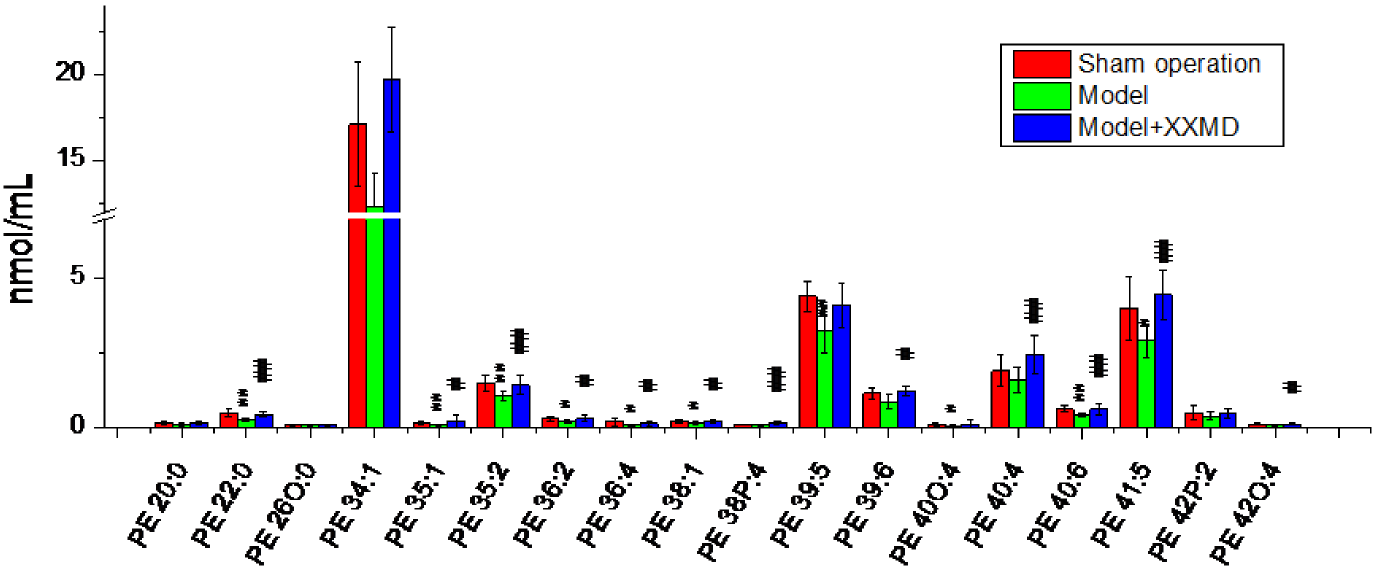
Sham operation
Model
Model+XXMD
nmol/mL
PE 20:0
PE 22:0
PE 26O:0
PE 34:1
PE 35:1
PE 35:2
PE 36:2
PE 36:4
PE 38:1
PE 38P:4
PE 39:5
PE 39:6
PE 40O:4
PE 40:4
PE 40:6
PE 41:5
PE 42P:2
PE 42O:4

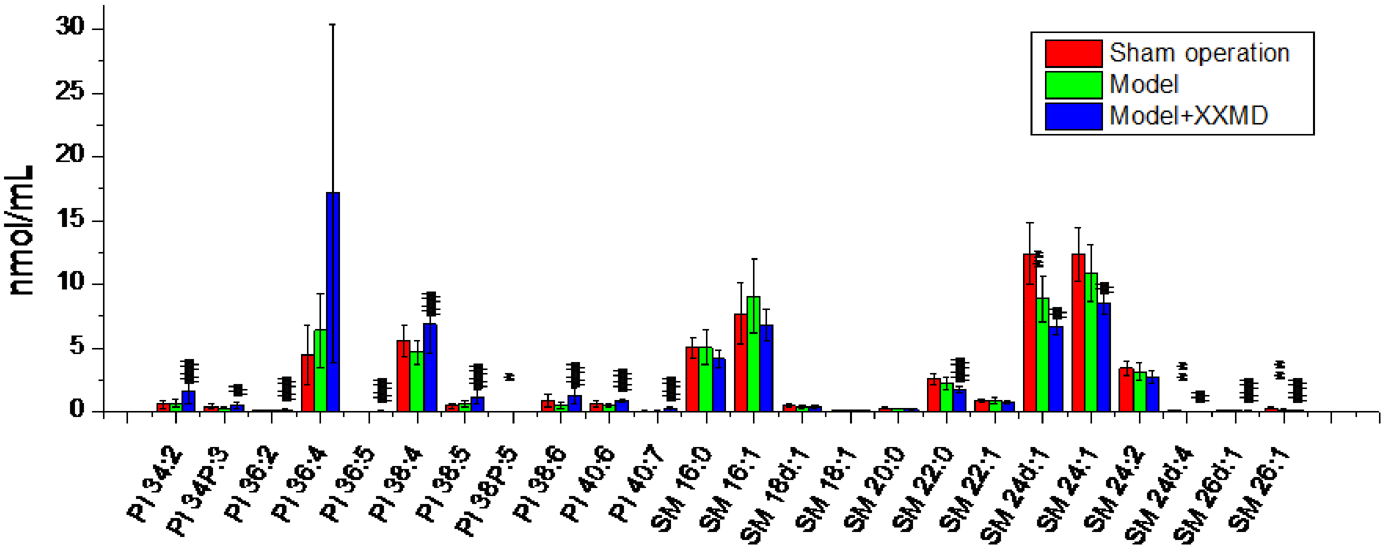
Sham operation
Model
Model+XXMD
nmol/mL
PI 34:2
PI 34P:3
PI 36:2
PI 36:4
PI 36:5
PI 38:4
PI 38:5
PI 38P:5
PI 38:6
PI 40:6
PI 40:7
SM 16:0
SM 16:1
SM 18d:1
SM 18:1
SM 20:0
SM 22:0
SM 22:1
SM 24d:1
SM 24:1
SM 24:2
SM 24d:4
SM 26d:1
SM 26:1

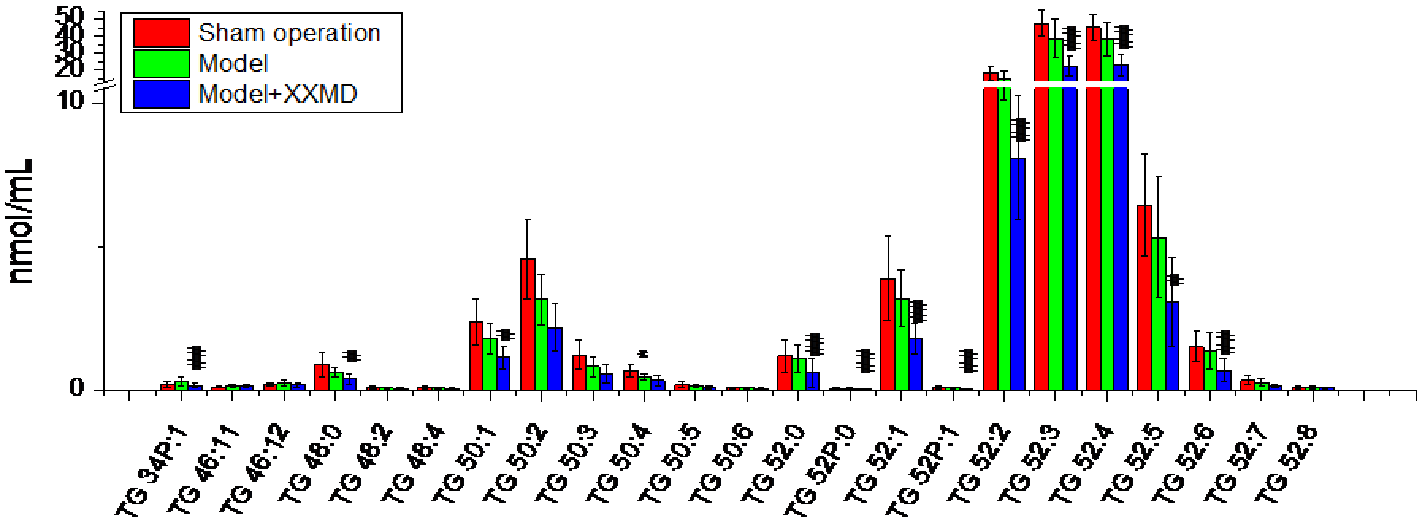
Sham operation
Model
Model+XXMD
nmol/mL
50
40
30
20
10
0
TG 34P:1
TG 46:11
TG 46:12
TG 48:0
TG 48:2
TG 48:4
TG 50:1
TG 50:2
TG 50:3
TG 50:4
TG 50:5
TG 50:6
TG 52:0
TG 52P:0
TG 52:1
TG 52P:1
TG 52:2
TG 52:3
TG 52:4
TG 52:5
TG 52:6
TG 52:7
TG 52:8

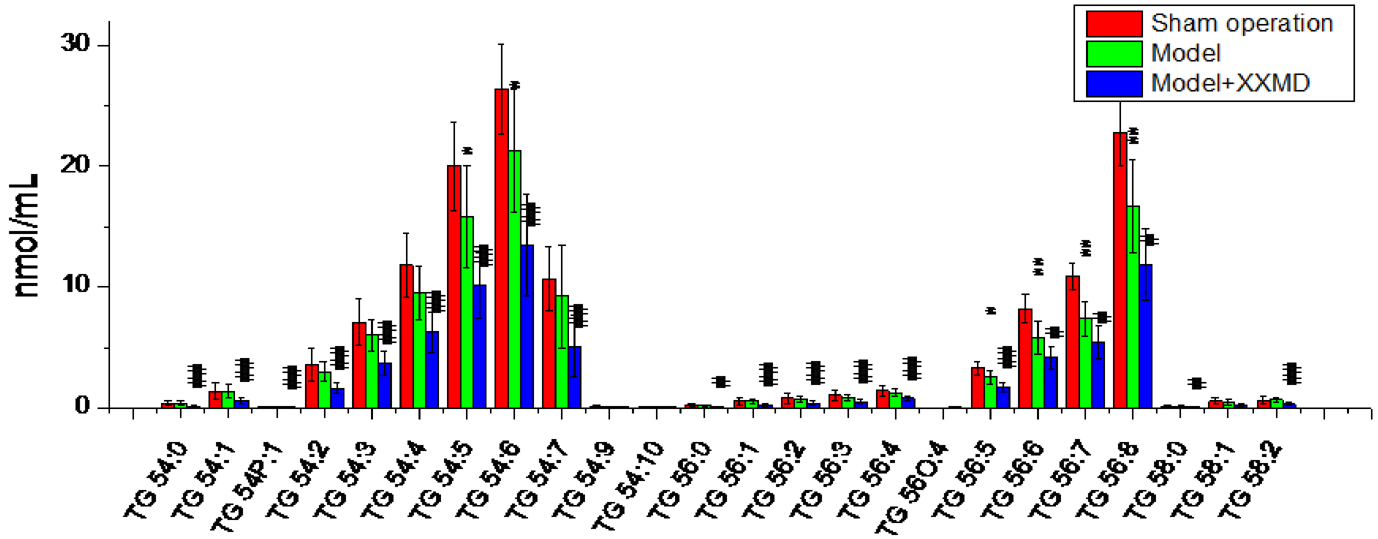
Sham operation
Model
Model+XXMD
nmol/mL
30
20
10
0
TG 54:0
TG 54:1
TG 54P:1
TG 54:2
TG 54:3
TG 54:4
TG 54:5
TG 54:6
TG 54:7
TG 54:9
TG 54:10
TG 56:0
TG 56:1
TG 56:2
TG 56:3
TG 56:4
TG 56O:4
TG 56:5
TG 56:6
TG 56:7
TG 56:8
TG 58:0
TG 58:1
TG 58:2

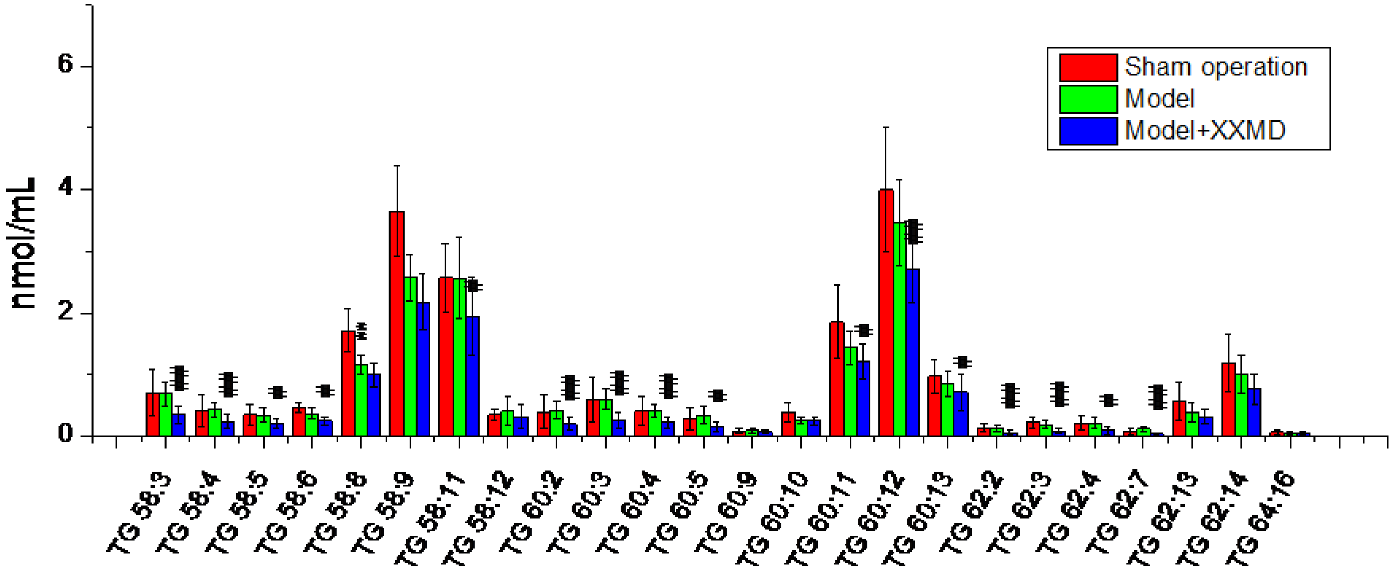
Sham operation
Model
Model+XXMD
nmol/mL
6
4
2
0
TG 58:3
TG 58:4
TG 58:5
TG 58:6
TG 58:8
TG 58:9
TG 58:11
TG 58:12
TG 60:2
TG 60:3
TG 60:4
TG 60:5
TG 60:9
TG 60:10
TG 60:11
TG 60:12
TG 60:13
TG 62:2
TG 62:3
TG 62:4
TG 62:7
TG 62:13
TG 62:14
TG 64:16

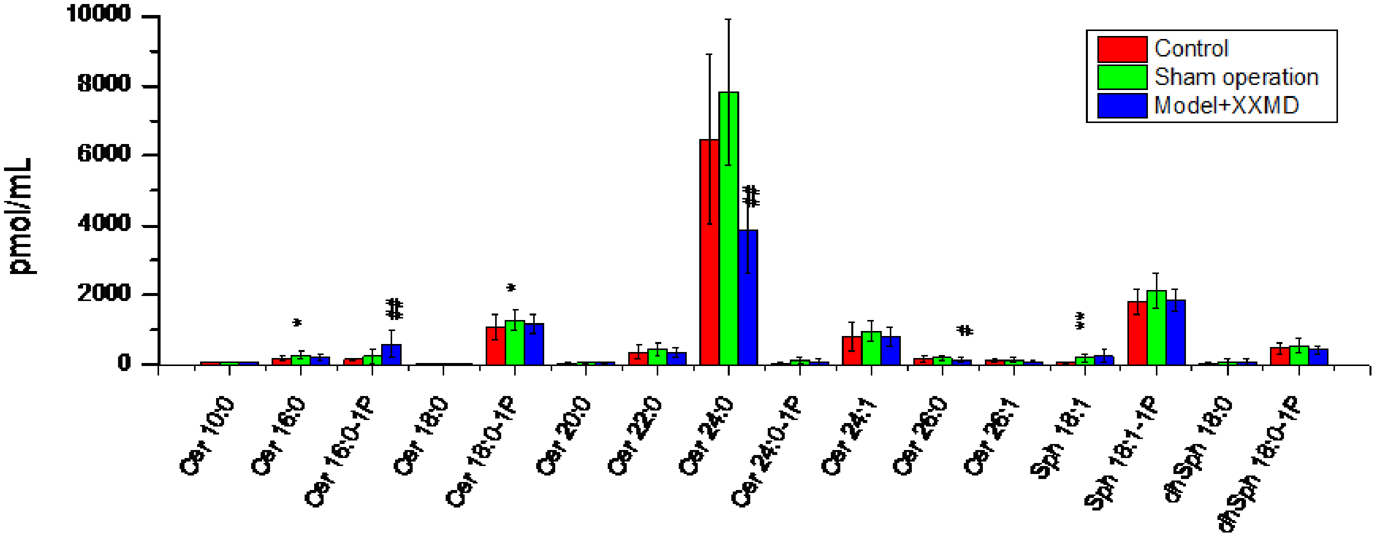

10000
8000
6000
4000
2000
0
pmol/mL
Control
Sham operation
Model+XXMD
Cer 10:0
Cer 16:0
Cer 16:0-1P
Cer 18:0
Cer 18:0-1P
Cer 20:0
Cer 22:0
Cer 24:0
Cer 24:0-1P
Cer 24:1
Cer 26:0
Cer 26:1
Sph 18:1
Sph 18:1-1P
dhSph 18:0
dhSph 18:0-1P

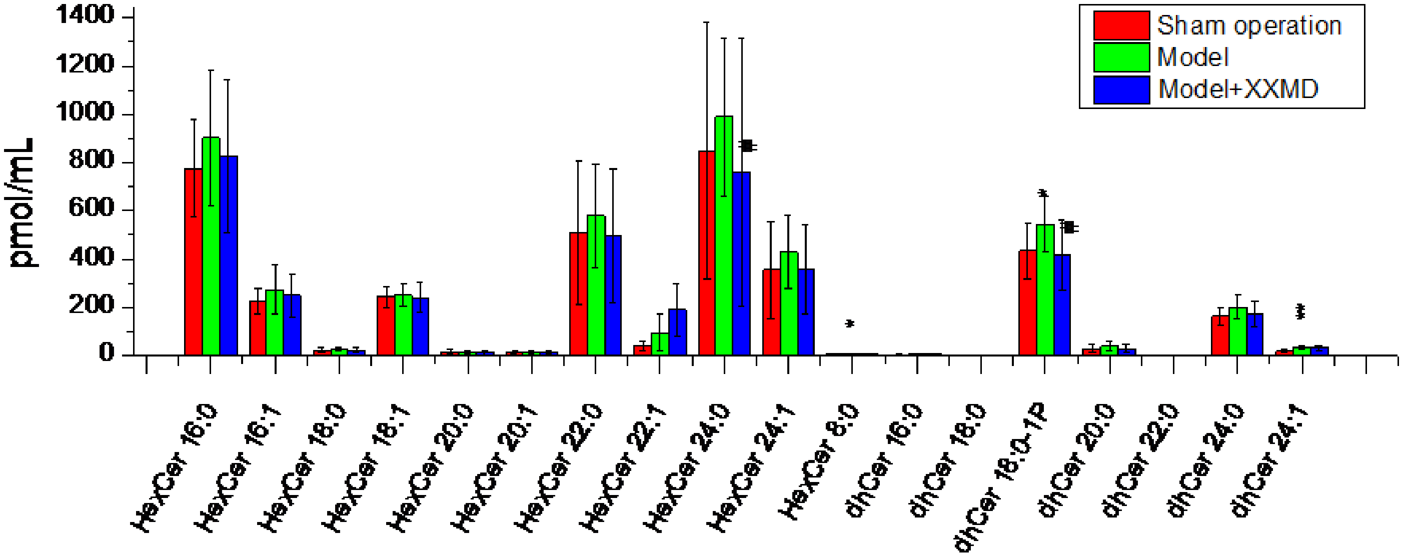

1400
1200
1000
800
600
400
200
0
pmol/mL
Sham operation
Model
Model+XXMD
HexCer 16:0
HexCer 16:1
HexCer 18:0
HexCer 18:1
HexCer 20:0
HexCer 20:1
HexCer 22:0
HexCer 22:1
HexCer 24:0
HexCer 24:1
HexCer 8:0
dhCer 16:0
dhCer 18:0
dhCer 18:0-1P
dhCer 20:0
dhCer 22:0
dhCer 24:0
dhCer 24:1

Figure S5. Bar graphs of compounds' concentration detected in brain tissue. (* stands for comparison with sham operation group, # stands for comparison with model group. Single symbol means P<0.05 while double means P<0.01)

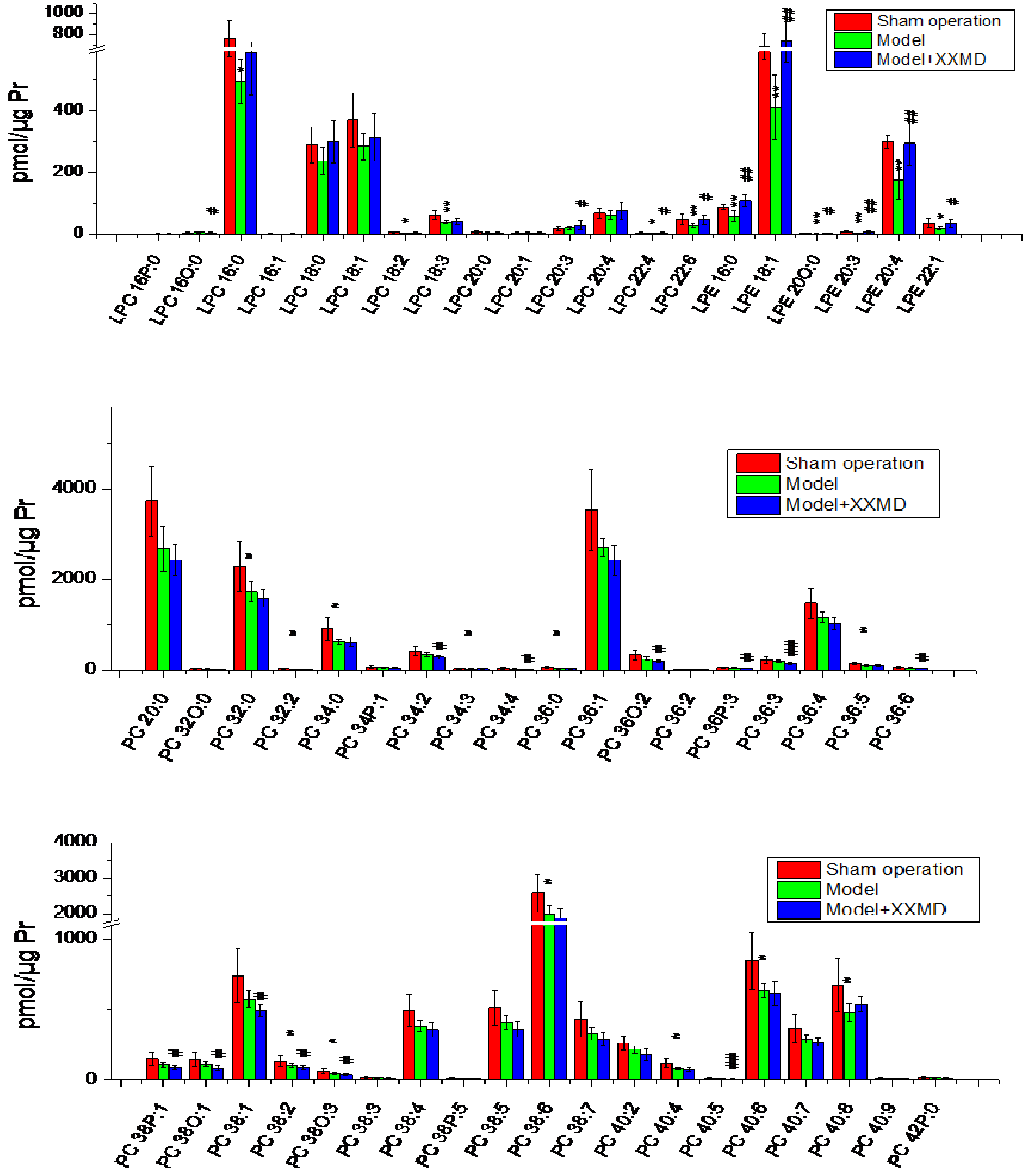

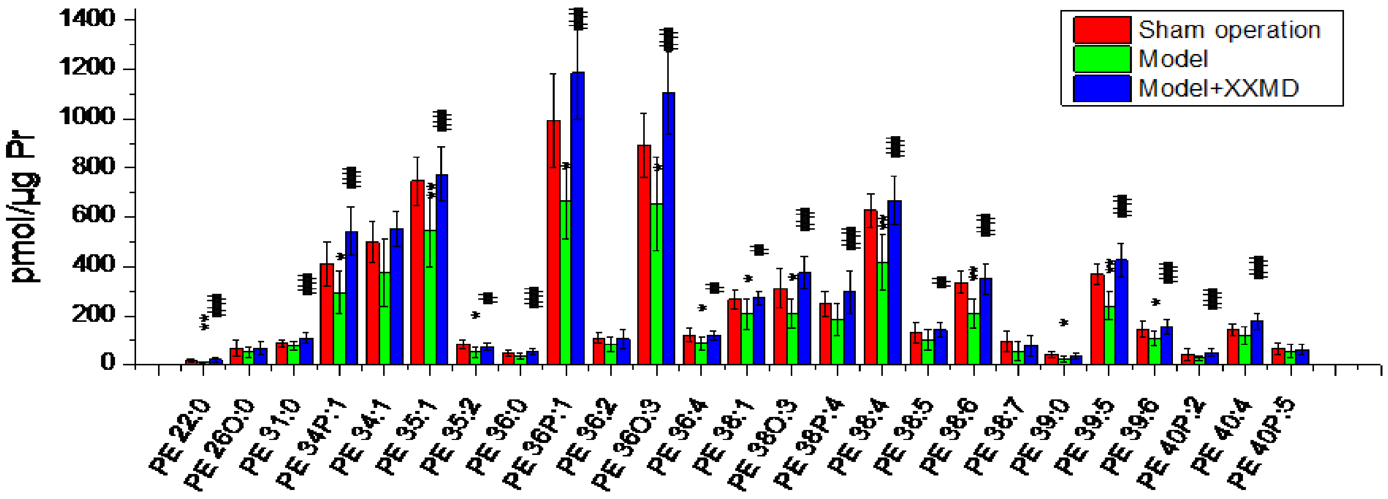

pmol/µg Pr
0
200
400
600
800
1000
1200
1400
Sham operation
Model
Model+XXMD
PE 22:0
PE 26O:0
PE 31:0
PE 34P:1
PE 34:1
PE 35:1
PE 35:2
PE 36:0
PE 36P:1
PE 36:2
PE 36O:3
PE 36:4
PE 38:1
PE 38O:3
PE 38P:4
PE 38:4
PE 38:5
PE 38:6
PE 38:7
PE 39:0
PE 39:5
PE 39:6
PE 40P:2
PE 40:4
PE 40P:5

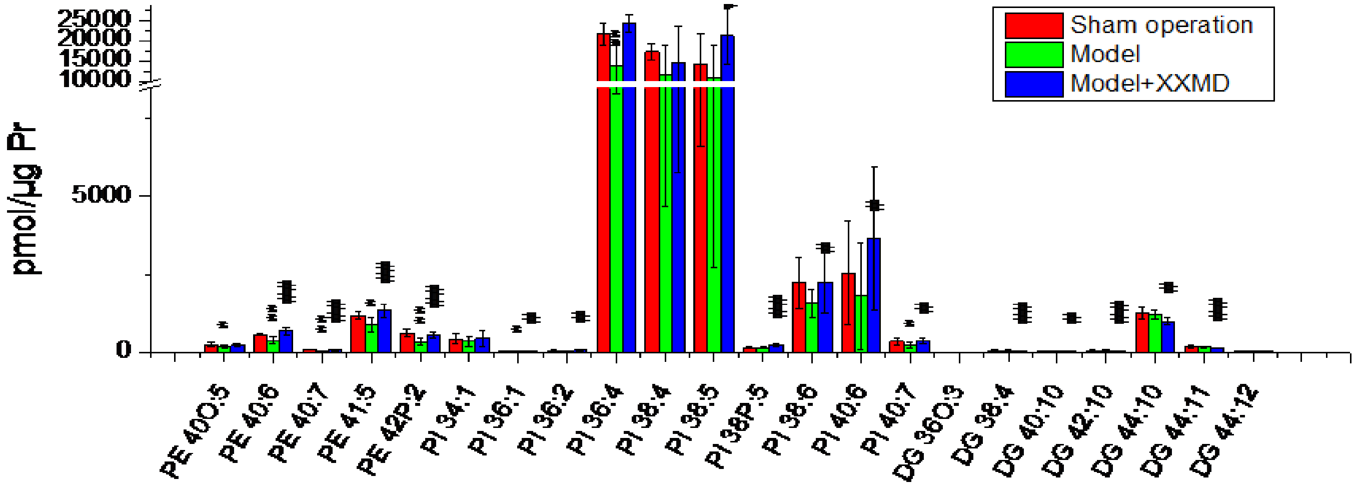

pmol/µg Pr
0
5000
10000
15000
20000
25000
Sham operation
Model
Model+XXMD
PE 40O:5
PE 40:6
PE 40:7
PE 41:5
PE 42P:2
PI 34:1
PI 36:1
PI 36:2
PI 36:4
PI 38:4
PI 38:5
PI 38P:5
PI 38:6
PI 40:6
PI 40:7
DG 36O:3
DG 38:4
DG 40:10
DG 42:10
DG 44:10
DG 44:11
DG 44:12

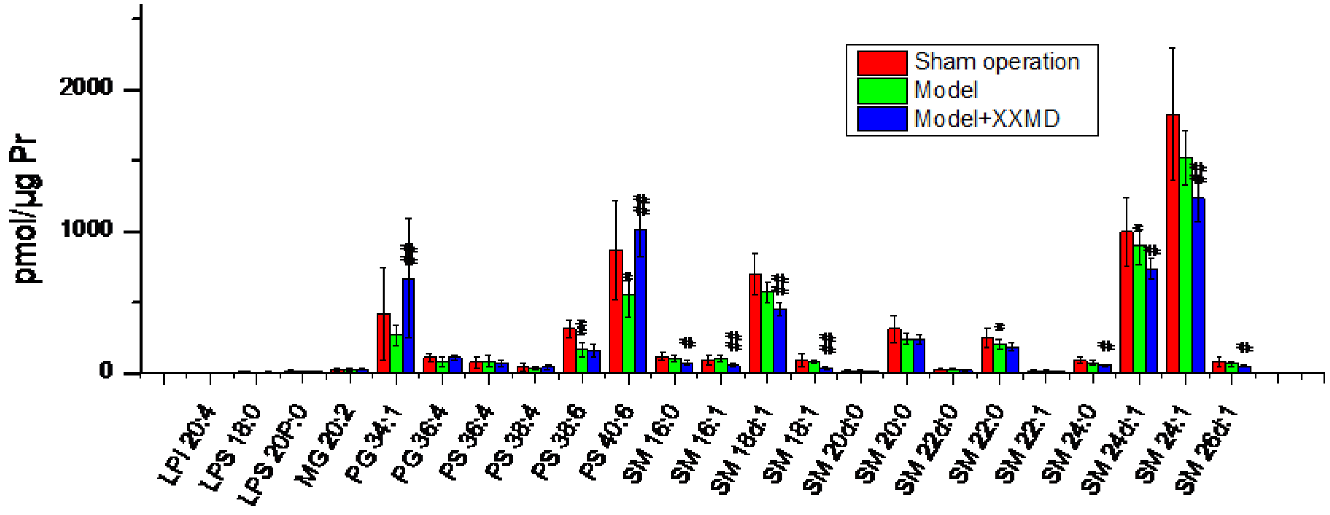

pmol/µg Pr
0
1000
2000
Sham operation
Model
Model+XXMD
LPI 20:4
LPS 18:0
LPS 20P:0
MG 20:2
PG 34:1
PG 36:4
PS 36:4
PS 38:4
PS 38:6
PS 40:6
SM 16:0
SM 16:1
SM 18d:1
SM 18:1
SM 20d:0
SM 20:0
SM 22d:0
SM 22:0
SM 22:1
SM 24:0
SM 24d:1
SM 24:1
SM 26d:1

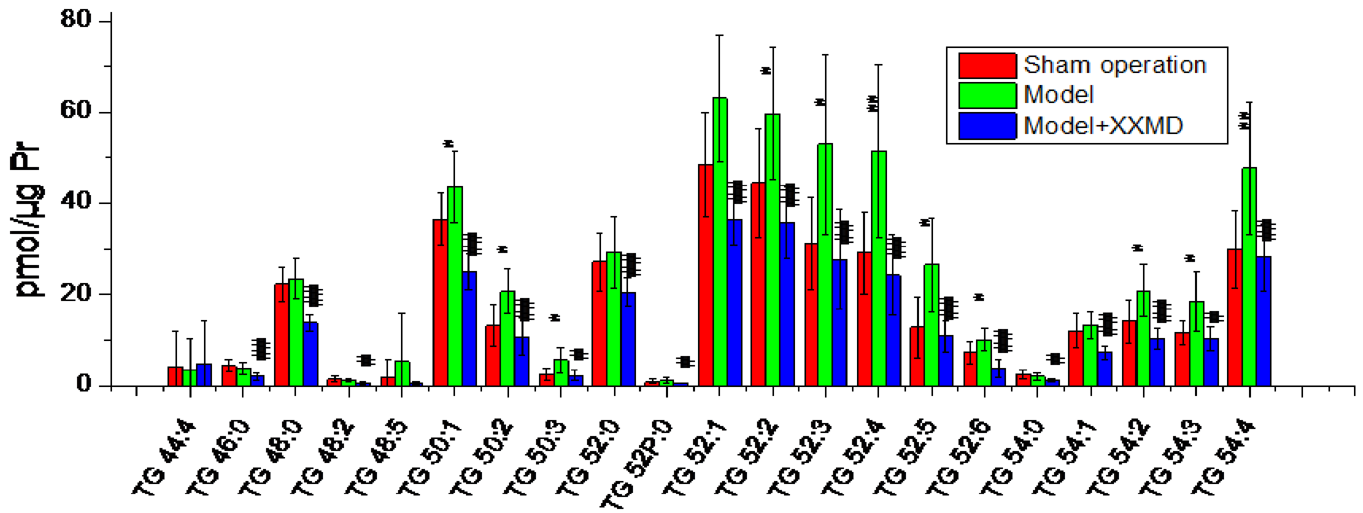
pmol/μg Pr
0
20
40
60
80
Sham operation
Model
Model+XXMD
TG 44:4
TG 46:0
TG 48:0
TG 48:2
TG 48:5
TG 50:1
TG 50:2
TG 50:3
TG 52:0
TG 52P:0
TG 52:1
TG 52:2
TG 52:3
TG 52:4
TG 52:5
TG 52:6
TG 54:0
TG 54:1
TG 54:2
TG 54:3
TG 54:4

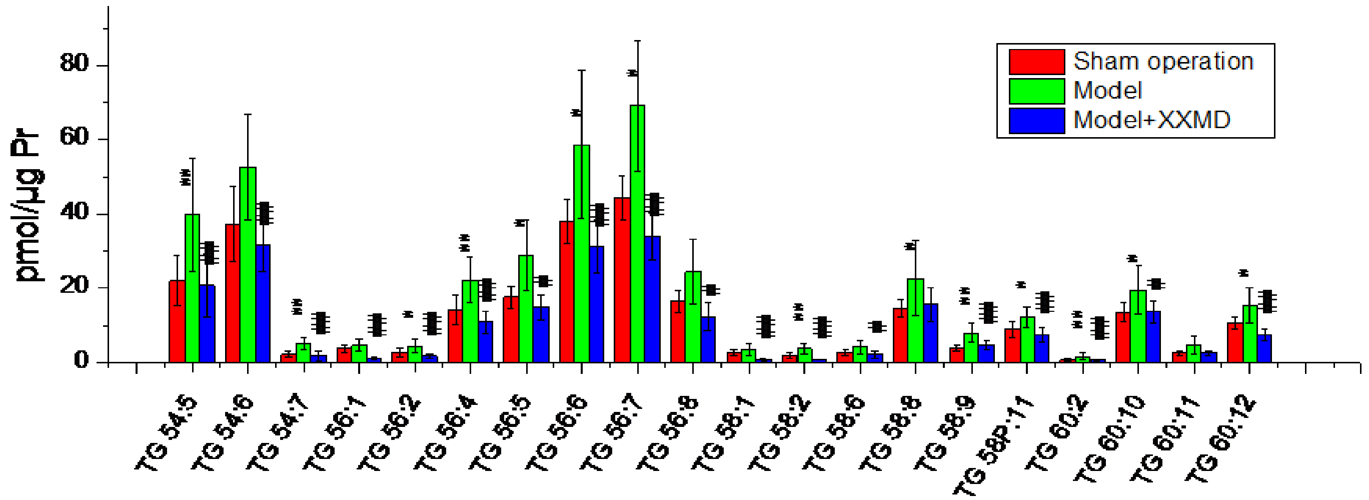
pmol/μg Pr
0
20
40
60
80
Sham operation
Model
Model+XXMD
TG 54:5
TG 54:6
TG 54:7
TG 56:1
TG 56:2
TG 56:4
TG 56:5
TG 56:6
TG 56:7
TG 56:8
TG 58:1
TG 58:2
TG 58:6
TG 58:8
TG 58:9
TG 58P:11
TG 60:2
TG 60:10
TG 60:11
TG 60:12

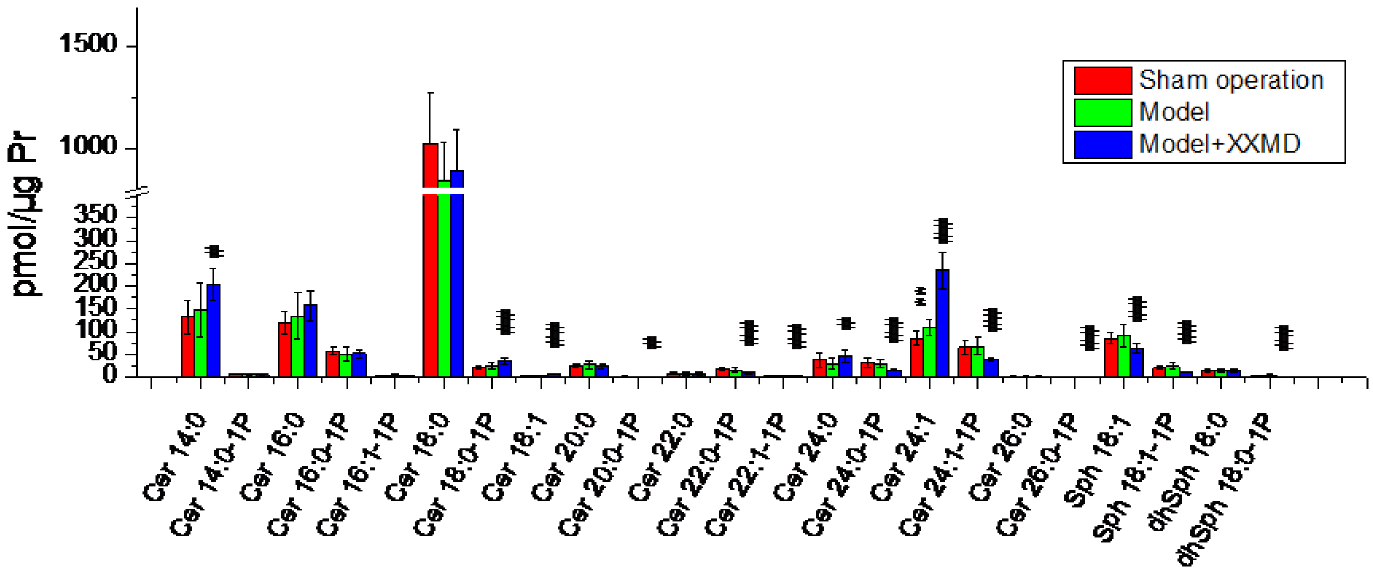
pmol/μg Pr
0
50
100
150
200
250
300
350
1000
1500
Sham operation
Model
Model+XXMD
Cer 14:0
Cer 14:0-1P
Cer 16:0
Cer 16:0-1P
Cer 16:1-1P
Cer 18:0
Cer 18:0-1P
Cer 18:1
Cer 20:0
Cer 20:0-1P
Cer 22:0
Cer 22:0-1P
Cer 22:1-1P
Cer 24:0
Cer 24:0-1P
Cer 24:1
Cer 24:1-1P
Cer 26:0
Cer 26:0-1P
Sph 18:1
Sph 18:1-1P
dhSph 18:0
dhSph 18:0-1P

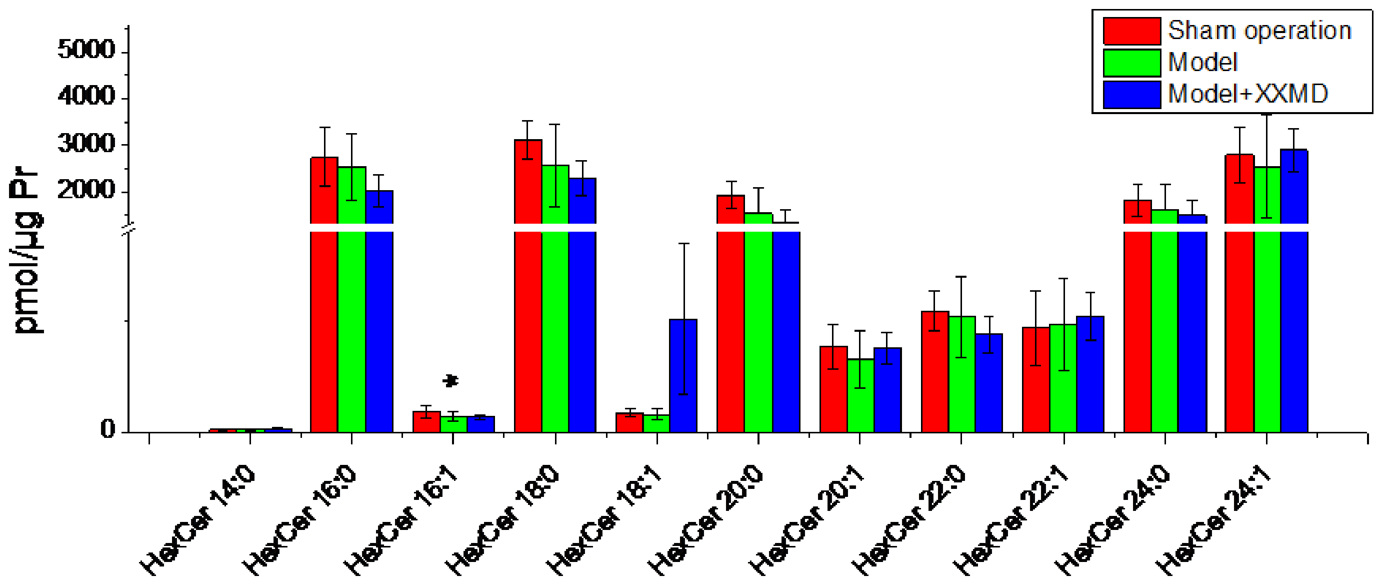
Sham operation
Model
Model+XXMD
pmol/μg Pr
5000
4000
3000
2000
0
HexCer 14:0
HexCer 16:0
HexCer 16:1
HexCer 18:0
HexCer 18:1
HexCer 20:0
HexCer 20:1
HexCer 22:0
HexCer 22:1
HexCer 24:0
HexCer 24:1

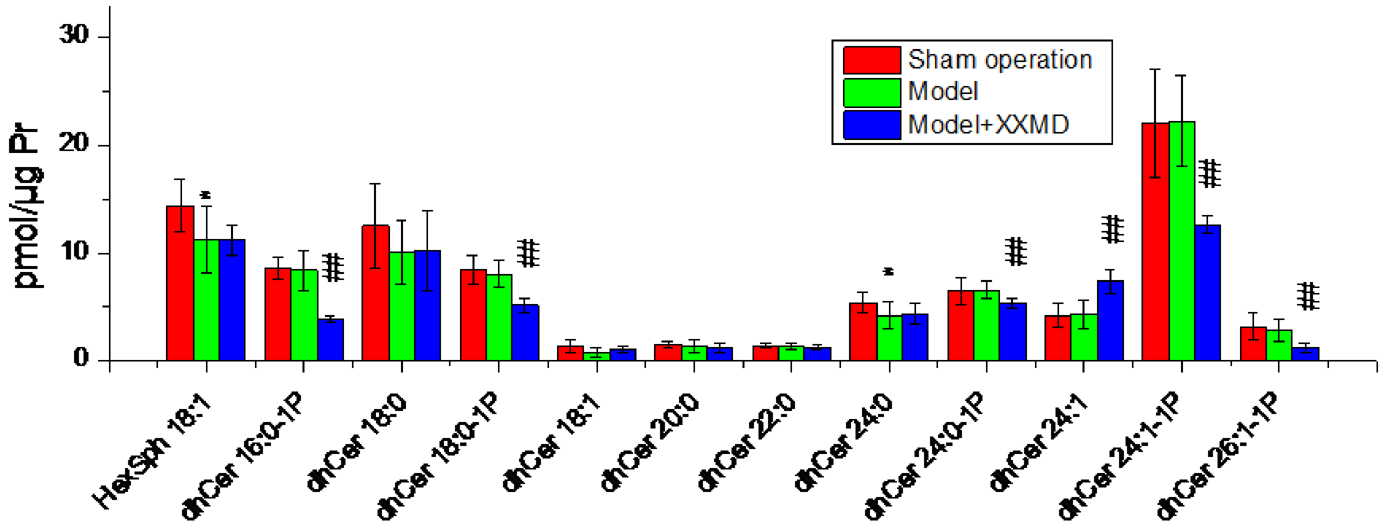
Sham operation
Model
Model+XXMD
pmol/μg Pr
30
20
10
0
HexSph 18:1
dhCer 16:0-1P
dhCer 18:0
dhCer 18:0-1P
dhCer 18:1
dhCer 20:0
dhCer 22:0
dhCer 24:0
dhCer 24:0-1P
dhCer 24:1
dhCer 24:1-1P
dhCer 26:1-1P

Figure S6. Structure framework of metabolites of XXMD after oral administration to rats

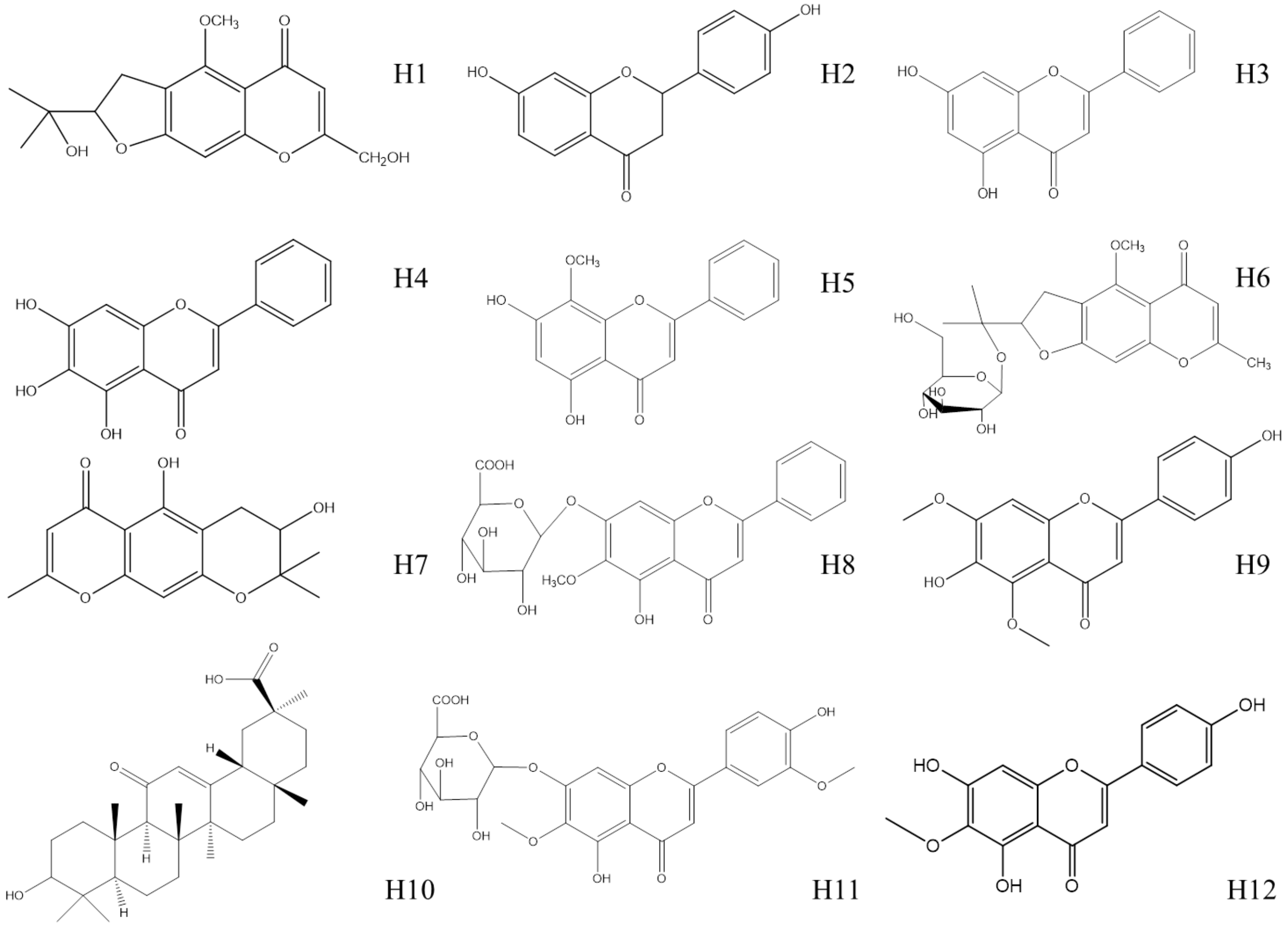

References

1. Matyash, V., Liebisch, G., Kurzchalia, T. V., Shevchenko, A. & Schwudke, D. 2008. Lipid extraction by methyl- tert- butyl ether for high- throughput lipidomics. *J Lipid Res*. **49**: 1137-1146.

2. Qu, F., Wu, C. S., Hou, J. F., Jin, Y. & Zhang, J. L. 2012. Sphingolipids as new biomarkers for assessment of delayed-type hypersensitivity and response to triptolide. *PLoS One*. **7**: e52454.

3. Fauland, A. *et al*. A comprehensive method for lipid profiling by liquid chromatography-ion cyclotron resonance mass spectrometry. *J Lipid Res*. **52**: 2314-2322.

4. Qu, F. *et al*. 2014. Lipidomic profiling of plasma in patients with chronic hepatitis C infection. *Anal Bioanal Chem*. **405**: 555-564.

5. Wang, C. H., Jia, Z. X., Wang, Z., *et al*. 2016. Pharmacokinetics of 21 active components in focal cerebral ischemic rats after oral administration of the active fraction of Xiao-xu-ming decoction. *J Pharm Biomed Anal*. **122**: 110-117.

6. Wang, C. H., Wu, C. S., Qin, H. L., et al. 2014. Rapid discovery and identification of 68 compounds in the active fraction from Xiao-Xu-Ming decoction (XXMD) by HPLC-HRMS and MTSF technique. *Chin Chem Lett.* **25**: 1648-1652.
